# Expanding the diversity of Chardonnay aroma through the metabolic interactions of *Saccharomyces cerevisiae* cocultures

**DOI:** 10.3389/fmicb.2022.1032842

**Published:** 2023-02-09

**Authors:** Fanny Bordet, Rémy Romanet, Florian Bahut, Jordi Ballester, Camille Eicher, Cristina Peña, Vicente Ferreira, Régis Gougeon, Anne Julien-Ortiz, Chloé Roullier-Gall, Hervé Alexandre

**Affiliations:** ^1^PAM UMR A 02.102, Univ. Bourgogne Franche-Comté, Institut Agro Dijon, IUVV, Dijon, France; ^2^Lallemand SAS, Blagnac, France; ^3^Centre des Sciences du Goût et de l’Alimentation, CNRS, INRAE, Institut Agro, Université Bourgogne Franche-Comté, Dijon, France; ^4^Dpt. Química Analítica, Facultad de Ciencias, University of Zaragoza, Zaragoza, Spain; ^5^DIVVA (Développement Innovation Vigne Vin Aliments) Platform/PAM UMR, IUVV, Dijon, France

**Keywords:** wine, nitrogen metabolism, yeast-yeast interaction, *Saccharomyces cerevisiae*, fermentation, metabolomics (OMICS), volatilome profiles, sensory analisys

## Abstract

Yeast co-inoculations in winemaking are often studied in the framework of modulating the aromatic profiles of wines. Our study aimed to investigate the impact of three cocultures and corresponding pure cultures of *Saccharomyces cerevisiae* on the chemical composition and the sensory profile of Chardonnay wine. Coculture makes it possible to obtain completely new aromatic expressions that do not exist in the original pure cultures attributed to yeast interactions. Esters, fatty acids and phenol families were identified as affected. The sensory profiles and metabolome of the cocultures, corresponding pure cultures and associated wine blends from both pure cultures were found to be different. The coculture did not turn out to be the addition of the two pure culture wines, indicating the impact of interaction. High resolution mass spectrometry revealed thousands of cocultures biomarkers. The metabolic pathways involved in these wine composition changes were highlighted, most of them belonging to nitrogen metabolism.

## 1. Introduction

Microorganisms coexist in complex consortia within the various ecosystems of our environment. This coexistence is now known to involve interactions that can be positive, negative, or neutral. Many ecological niches have been described as hosting these consortia, including wine. A large diversity of microorganisms is found on the grape berry (Fleet, 2003; Barata et al., 2012) which decreases during the winemaking process. This loss of diversity is induced both by environmental conditions and by the interactions between microorganisms. Also, during alcoholic fermentation, the fermentative yeasts predominate and will coexist. This cohabitation gives rise to different types of interactions and has been widely studied in recent years (Comitini et al., 2021; Zilelidou and Nisiotou, 2021). Until now, most studies have focused on interactions in the context of two microorganism cocultures, although studies of more complex consortia are emerging (Conacher et al., 2020). Different mechanisms of interactions and their impact on wine composition have been described (Bordet et al., 2020). These interactions were often found to be non-neutral, leading to a change of the metabolism and/or growth of one or both populations. In general, studies have focused on the interactions between *Saccharomyces* and non-*Saccharomyces* yeasts for various applications such as avoiding the risks of stuck fermentations, improving the specific sensory profiles of products, ethanol reduction or the control of spontaneous or unwanted microorganisms (Ciani et al., 2010). However, information on the interactions between yeasts within the same genus or species is limited. Interactions were highlighted between a *Saccharomyces cerevisiae* strain and a *Saccharomyces bayanus* strain, and between two *S. cerevisiae* strains by comparing cocultures to pure cultures and mixing wines resulting from the fermentation of isolated yeasts (Howell et al., 2006; King et al., 2008; Capece et al., 2013). These mechanisms can be studied using different approaches, including specific metabolite quantification, and the assessment of nutrient uptake and consumption (Rollero et al., 2018; Petitgonnet et al., 2019; Scott et al., 2021). Nevertheless, the monitoring of population dynamics remains essential to understand the types of interactions and give insights into the mechanisms involved (Bordet et al., 2020). Moreover, between yeasts of the same genus or species, discriminating the populations involved remains a complex step. It is necessary to find keys to lift this technological barrier in both classical culture techniques and flow cytometry enumeration. In flow cytometry, yeast fluorescent labeling is increasingly used to study cocultures or more complex consortia (Petitgonnet et al., 2019; Conacher et al., 2020). In addition to cell counting, this technique is used to obtain information on the physiological state of cells during monitoring.

Furthermore, other approaches are used to determine the impact of coculture on genome expression, its regulation and yeast metabolism. Indeed, new omics technologies including proteomics (Peng et al., 2019) and transcriptomics (Curiel et al., 2017) have been applied to the study of these interactions. However, metabolomics, used in our study, provides information well beyond cell phenotype according to genome and its expression under specific environmental conditions, offering an overview of possible biochemical regulations. A phenotypic representation of microorganisms was given in accordance with their genetic background, regulation, and expression under environmental changes (Pinu, 2018). Recently, ultrahigh resolution mass spectrometry (uHRMS) has revealed interactions between *S. cerevisiae* and non-*Saccharomyces* (Petitgonnet et al., 2019; Roullier-Gall et al., 2020) Differences in wine composition were demonstrated when comparing cocultures involving a strain of *S. cerevisiae* and *Lachancea thermotolerans, Starmerella bacillaris, Metschnikowia pulcherrima* with pure cultures, showing the presence of interactions. The wine chemical composition obtained from cocultures appeared to differ from the addition of the two metabolomes of single strains (Roullier-Gall et al., 2020). Moreover, a specific interaction mechanism was revealed by the study, with or without cell–cell contact between the two-yeast species. Indeed, cell–cell contact between *S. cerevisiae* and *L. thermotolerans* induces the production of new metabolites and quantitative metabolite changes (Petitgonnet et al., 2019). The use of this technique provides new insights, but this is only part of the picture when characterizing micro-organisms and their interaction more precisely.

Besides changes in primary metabolism, many studies have reported modulations of the composition of the volatilome in the context of coculture involving *S. cerevisiae* and non-*Saccharomyces*. Several families of volatile compounds were described as being involved, such as esters, higher alcohols, and fatty acids (Fleet, 2003; Bordet et al., 2020). Morales et al., 2019 showed that 2-phenylethyl acetate and isoamyl acetate concentrations increased when combining *L. thermotolerans* and *S. cerevisiae* compared to pure *S. cerevisiae* fermentation conditions. This was also observed in the context of the coculture of *T. delbrueckii* and *S. cerevisiae* by Renault et al. (2015). Similarly, coculture can increase the higher alcohol content of wines. This was detailed by Sadoudi et al. (2012) for mixed fermentations with *Candida zemplinina* and *S. cerevisiae* and also for *T. delbrueckii* and *S. cerevisiae*. Medium chain fatty acids were found mostly decreased in coculture compared to pure culture, as observed by Nardi et al. (2019). The sensory aspect was also addressed by researchers but few of them linked it to volatile compounds and interactions (Zhang et al., 2018). Changes at the sensory level have been highlighted for wines obtained from cocultures, thereby confirming interactions between microorganisms (Fleet, 2003). However, most studies have focused on the interactions between *S. cerevisiae* and non-*Saccharomyces*.

These aromatic volatile compounds modifications of wine resulting from interaction phenomena may be a way to modify the sensory profile of wine. Furthermore, the majority of studies on these interactions focus on a set of different cocultures of strains, and often revolve around the global comparison of these sets of cocultures with their corresponding pure cultures. Thus, interactions are not sufficiently detailed and discussed from the perspective of a given coculture versus the associated single strains, therefore not allowing for in depth establishment of differences between the two cultivation modes for these given strains. An integrative approach combining different targeted and non-targeted approaches is essential (Pinu, 2018; Bordet et al., 2021). Therefore, in this study, we were interested in determining whether this type of interaction existed within the same species, in this case *S. cerevisiae*. We investigated for the first-time interactions occurring between different *S. cerevisiae* strains under coculture using an integrative approach.

## 2. Materials and methods

### 2.1. *Saccharomyces cerevisiae* strains

A total of five strains of *S. cerevisiae* were used in this study. Four commercial strains of *S. cerevisiae* (Lallemand Inc., Montreal, QC, Canada) were selected from among the strains considered in our previous work (Bordet et al., 2021). These commercial strains were coded S2, S3, S4 and S8, supplied as active dry yeast (ADY) and stored at 4°C once opened. The other strain, *S. cerevisiae* S3GFP was a modified *S. cerevisiae* strain supplied from the VAlMiS team, at the Burgundy University Vine and Wine Institute. S3GFP is a derivative of the commercial strain S3 modified by CRIPSR-Cas 9 to strongly express e*EGFP* (Bordet et al., 2022). This modified strain was stored at – 80°C in YPD liquid medium [0.5% w/v yeast extract (Biokar, Beauvais, France), 1% w/v bactopeptone (Biokar), 2% w/v D-glucose (Prolabo, Fontenay sous-Bois, France) and 0.02% w/v chloramphenicol (Sigma, St Louis, MI, United States)], containing 20% v/v glycerol.

### 2.2. Growth conditions

The modified strain was isolated on YPD plate (YPD liquid with 2% w/v agar (Biokar, Pantin, France)) at 28°C. One colony was added in YPD liquid medium at 28°C for 24 h. Following the rehydration of commercial yeast and the suspension of modified yeast, all the strains were grown as previously described (Bordet et al., 2021). Briefly, each strain was diluted at 0.1% (v/v) in 250 ml sterile Erlenmeyer flasks containing 150 ml of modified YPD medium and closed with dense cotton plugs. After incubation for 18 h at 28°C under agitation (150 rpm), the second culture, in 150 ml of pasteurized Chardonnay must, filtered on a 0.22 μm membrane (Steritop-GP, MERCK-Millipore, Burlington, Massachusetts, United States), was performed in Erlenmeyer flasks (250 ml) at 28°C without stirring for 18 h.

### 2.3. Fermentation conditions

Fermentations were carried out in three biological replicates in pasteurized Chardonnay must containing 226 g.l^−1^ glucose/fructose, pH 3.92, and 343 mg.l^−1^ total assimilable nitrogen.

Pure fermentations and co-fermentations were conducted in 2 l sterile bottles containing 1 l of Chardonnay must and closed with sterile cotton wool. Each fermentation was conducted at 20°C in static mode. The end of fermentation was considered as the total depletion of sugars.

Pure fermentations were inoculated with the latter must cells cultures at 1 × 10^6^ viable cells.ml^−1^. Co-fermentations were inoculated by two *S. cerevisiae* as explained in Supplementary Table S1. Each strain was inoculated at 5 × 10^5^ viable cells.ml^−1^, also from Chardonnay must cultures. Daily sampling was carried out.

**Figure 1:**
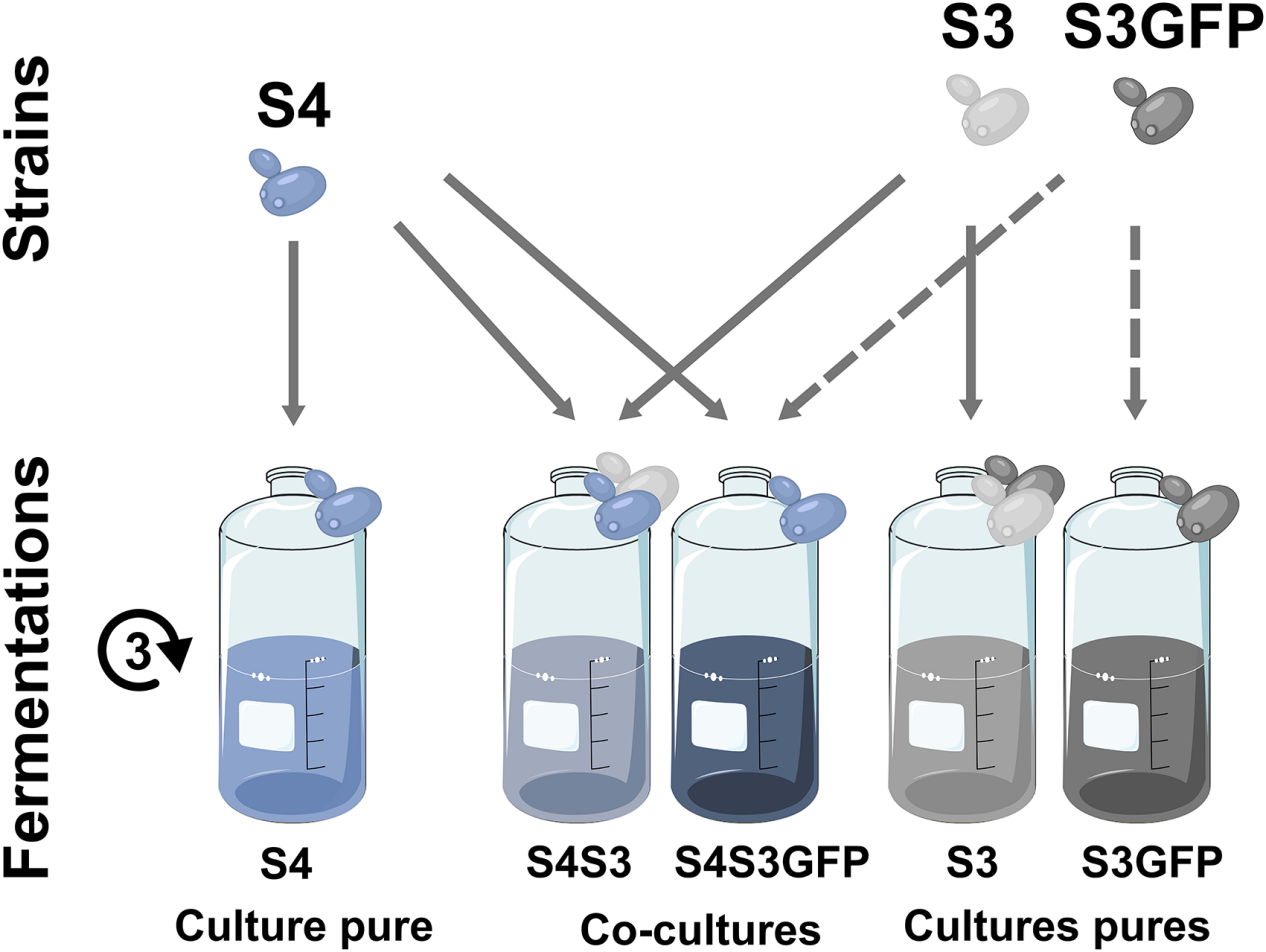
Experimental design. Experimental design and sample generation for the S4S3 modality.

We compared pure cultures of three strains of *S. cerevisiae* and cocultures, combining each of the three commercial strains (S2, S4, S8) with one EGFP-labelled *S. cerevisiae* (S3GFP) or its corresponding wild-type strain (S3). For each of the approaches used, the results are presented for each of the modalities (S2S3, S4S3 and S8S3) under study according to the three corresponding fermentations: pure cultures of each strain, cocultures of the commercial strain with the S3 strain and cocultures of the commercial strain with the S3GFP strain (Figure 1).

### 2.4. Growth monitoring

Cell viability in preculture and during fermentation was determined by flow cytometry. Dissolved propidium iodide (PI; Invitrogen, Molecular Probes, ThermoFisher Scientific, Illkrich, France) in water at a concentration of 0.1 μg.ml^−1^ was used as fluorochrome. We added 1 μl of PI to 100 μl of diluted suspension in PBS buffer (137 mM NaCl, 2.7 mM KCl, and 11.9 mM Phosphate, pH 7.2; Fisher Scientific, Illkirch, France). Samples were incubated in the dark for 15 min before analysis. Flow cytometry was performed with a BD Accuri C6 flow cytometer (Becton, Dickinson and Company Franklin Lakes, United States), and the data were processed using the BD Accuri C6 software. For each sample, 20 μl were analyzed at 34 μl.min^−1^. The FSH threshold used was 80,000. A 488-nm wavelength argon laser was used to excite the cells (autofluorescence). GFP fluorescence was assessed on the FL1-H band pass filter (533/530 nm), PI fluorescence was measured on the FL3-H long pass filter (675 nm) and side-scatter light (SSC)/fluorescence intensity data were analyzed.

### 2.5. Growth kinetics parameters and *Saccharomyces cerevisiae* fitness advantage

An assessment of the growth kinetics parameters of strains in pure and coculture were performed. The maximum growth rate (μ max), maximal population (K) and generation number to reach maximal population were determined. The fitness advantage (*m*) between two microorganisms can be defined, as developed by García-Ríos et al. (2014), by the following expression: *m* = *r*1 – *r*2, where *r*1 corresponds to the μmax for strain 1, *r*2 to the μmax for the strain 2 and m to the μmax difference between the two strains, under specific environmental conditions. In this study, we generated the m value with the comparison of each strain in coculture, for example *m* = *μ*maxS4 − *μ*max*S3GFP*.

A *t*-test was performed to compare each parameter between pure culture and coculture conditions for each modality. An ANOVA was also performed on the data by modality, followed by the Tukey HSD *post-hoc* test to characterize the differences between conditions, using R software (R-4.0.4).

### 2.6. Analytical methods

#### 2.6.1. Oenological analysis

Samples corresponding to three biological replicates were centrifuged at 8,000 ×*g* for 5 min at 4°C. Sugar concentration and ethanol degree were monitored daily by FTIR (Fourier-transformed infrared) spectroscopy (OenoFOSS™, FOSS, Hilleroed, Denmark). Fermentation kinetics could be established as sugar concentration against time. The maximal rate of sugar degradation and time to degrade 50% of the initial fermentable sugars were determined graphically. A *t*-test was performed to compare these two parameters between pure culture and coculture conditions in each modality and an ANOVA (value of *p* ≤ 0.05) was also performed followed by a Tukey HSD test on the data by modality (S2S3, S4S3, and S8S3) using R software (R-4.0.4).

#### 2.6.2. Volatilome analysis

Volatile compounds were determined by high performance gas chromatography. The major compounds were determined using the method developed for major volatile compounds based on the gas chromatographic-flame ionization detection analysis (GC-FID) of dichloromethane microextracts, as developed previously (Ortega et al., 2001). Briefly, 3 ml of wine were placed in 15 ml screw-capped centrifuge tubes containing 4.5 g of (NH_4_)_2_SO_4_, 7 ml of water, 15 μl of internal standard solution C (2-butanol, 4-methyl-2-pentanol, 4-hydroxy-4-methyl-2-pentanone and 2-octanol at 140 mg.ml^−1^ in ethanol) and 0.2 ml of dichloromethane. The samples were shaken and centrifuged. The dichloromethane phase was recovered and transferred in a vial for analysis. Analytes were separated on a DB-20 column from J&W Scientific (Folsom, CA, United States), with a length of 50 m, an internal diameter of 0.32 mm and a film thickness of 0.5 μm. The column temperature was held at 40°C for 5 min, then increased to 200°C at a rate of 3°C.min^−1^. The injection was fixed for 3 μl in split flow at a rate of 30 ml.min^−1^. Dihydrogen at a flow rate of 3 ml.min^−1^ was used as the carrier gas. Detection was by FID. The concentration of each specific compound was determined by computing the relative response areas to the corresponding internal standard. The measured relative area was then interpreted using the calibration graphs obtained by analyzing synthetic wines (12% (v/v) ethanol, 5 g.l^−1^ tartaric acid and pH adjusted to 3.2 with 1 M sodium hydroxide) containing known amounts of the volatile compounds.

The minor and trace volatile compounds were analyzed by solid-phase extraction (SPE) and gas chromatography with mass spectrometric detection (GC–MS) following the method described by López et al. (2002). This fraction was analyzed with a Star 3,400 CX gas chromatograph coupled to a Saturn 4 electronic impact ion trap mass spectrometer (Varian). Before analysis, the metabolites were separated on a DB-WAXetr (J&W, Folsom, United States) with a length of 60 m, an internal diameter of 0.25 mm and a film thickness of 0.5 μm and preceded by a 30 m × 0.32 mm uncoated precolumn. The oven temperature was held at 40°C for 5 min, then increased to 230°C at a rate of 2°C.min^−1^. The carrier gas was helium at 1 ml.min^−1^. 3 μl of samples after extraction by SPE (Vac ELUT 20 station from Varian) was injected in a 1,093 septum equipped programmable injector (SPI; Varian). The initial temperature of this injector was 30°C for 0.6 min and then raised to 230°C at 200°C.min^−1^. The extracted ion chromatogram was taken for comparison with the chemical standards and quantification by peak area.

The two methods were performed on the three biological replicates of each modality. Volatile compound quantification data were processed by performing an ANOVA (value of *p* < 0.05) followed by a Tukey test. All the results were processed using R software (R-4.0.4).

#### 2.6.3. Non-volatile metabolome analysis

The three biological wine replicates were analyzed by ultra-high performance liquid chromatography (Dionex Ultimate 3,000, ThermoFischer, Waltham, MA, United States) coupled to a MaXis plus MQESI-Q-ToF mass spectrometer (Bruker, Bremen, Germany). Nonpolar compounds were separated by reverse phase liquid chromatography (RP-LC) on an Acquity UPLC BEH C18 1.7 m column 100 × 2.1 mm (Waters, Guyancourt, France). Solvent A (5% (v/v) acetonitrile with 0.1% (v/v) formic acid) and solvent B (acetonitrile with 0.1% (v/v) formic acid) constituted the mobile phase used to elute the metabolites according to the following gradient: 5% (v/v) solvent B from 0 to 1.10 min followed by a linear increase in the proportion of solvent B from 1.10 to 6.40 min to reach 100% of the latter for 3.6 min, with a constant flow rate of 0.4 ml.min^−1^ maintained during the analysis. Ionization was implemented in negative and positive mode with an electrospray ionization source at a nebulization pressure of 2 bar and a dry nitrogen flow of 10 l.min^−1^. The mass spectrometer parameters were as follows: ion transfer (end plate offset at 500 V), capillary voltage (at 4500 V in positive ionization mode and at 3500 V in negative ionization mode), and acquisition (mass range 100–1,500 m/z). Fragmentation was realized at 8 Hz spectra rate using autoMS/MS function (20–50 eV).

Samples were centrifugated at 10,500 ×*g* for 10 min and preserved at 10°C during batch analysis. Before each batch analysis, Na formate cluster was injected directly into the source to perform an external calibration of the mass spectrometer. This calibration was carried out in “enhanced quadratic” mode with an error of less than 0.5 ppm. An internal calibration allowing a post-acquisition recalibration was also performed. The mass range was between 100 and 15,000 m/z. Inter-batch (standard peptide and polyphenol mixture) and intra-batch (experimental QC, sample mixture) quality controls were performed to ensure the repeatability and stability of the system during the analysis (Supplementary Figure S1). All the samples were randomly injected into the same batch to avoid batch-to-batch variability. The raw mass spectra of each sample were recalibrated using Compass DataAnalysis v4.3 software (Bruker, Bremen, Germany), to achieve a mass deviation after calibration of 0.5 ppm. The m/z associations and retention times (RT) also called features were extracted (S/N >30 and intensity thresholds >1,000). Features of each sample were aligned according to their RT and mass with a tolerance of less than 30 s and 10 ppm, respectively, using homemade R script: MXSAlign.^1^ Features were kept if they are present in more than 20% of the samples. The data resulting to positive and negative ionization mode were merged in one dataset, with tolerance, 5 ppm for m/z and 10 s for retention time. 5,191 features were obtained. Elementary formula was determined using isotopic profile (tolerance: 5 ppm and mSigma <20) and in house database. Only annotated features were selected for statistical analysis (2054 features). Once analyzed, Matlab (R202115a) was used for statistical analysis (PCA, *t*-test) and data visualization. Isolated significant features were annotated using the online tools of Metlin, KEGG, MassTrix, YMDB database and Oligonet.

#### 2.6.4. Descriptive sensory analysis

In order to conduct sensorial analysis at the end of alcoholic fermentation, the three biological replicates of each Chardonnay conditions were pooled to have enough volume per sample. Once pooled, the samples were filtered and sulfited at 40 mg.l^−1^ before bottling. The samples were stored in a cellar at constant temperature (16°C) for 6 months until sensory analysis.

Ten trained assessors were recruited for this study (four females and six males; median age, 40; range: 25–56). The ten assessors attended preliminary session to check their repeatability and their ability to recognize the typical attributes of Chardonnay wines. Sensory analyses took place in a sensory room equipped with individual booths. Each sample was assessed in ISO standardized black glasses covered with a plastic Petri dish and coded by a three-digit random number. Thirty milliliter of each wine was served at room temperature (20°C). The samples were assessed in a specific order for each panelist following a Latin square. For each sample, the assessors were asked to smell each samples and to rate the ortonasal intensity of following previously selected smell attributes to best describe the samples (translated from French): “white fruits,” “vanilla,” “yellow fruits,” “candied fruits,” “citrus,” “oxidation,” “pineapple,” “floral,” “amylic,” “polish,” “vegetal,” “reduction” on an eight-point scale ranging between “absent or missing” (score = 0) and “very strong/very intense” (score = 7) (Supplementary Figure S2). The sensory data were subjected to an ANOVA and Newman Keuls test (α = 0.05). Sensory statistical analyses were performed using R software (R-4.0.4).

## 3. Results and discussion

In this work we aimed to determine the influence of interactions between two *S. cerevisiae* yeast strains on yeast growth, fermentation kinetics, exometabolome, volatile compounds and sensory profile. Four strains were chosen since we previously reported that they possess specific metabolomic differences and lead to different wines after the alcoholic fermentation of Chardonnay must (Bordet et al., 2021). Our hypothesis is that the presence of two of these strains would also lead to wines that are different from those produced by pure cultures. For better and clearer understanding, the S4S3 modality will be discussed in detail in the present paper and compared to the two other modalities.

### 3.1. Growth and fermentation kinetics parameters

We first investigated the impact of the co-inoculation of two strains of *S. cerevisiae* on various phenotypic traits of growth and fermentation kinetics (Table 1). A previous study (Bordet et al., 2022) showed that the S3GFP strain and its corresponding wild type strain had identical growth and fermentation kinetics. Therefore, only the S3GFP strain will be considered in this section to follow it in coculture over time.

**Table 1:** Impact of coculturing on growth and fermentative kinetics parameters.

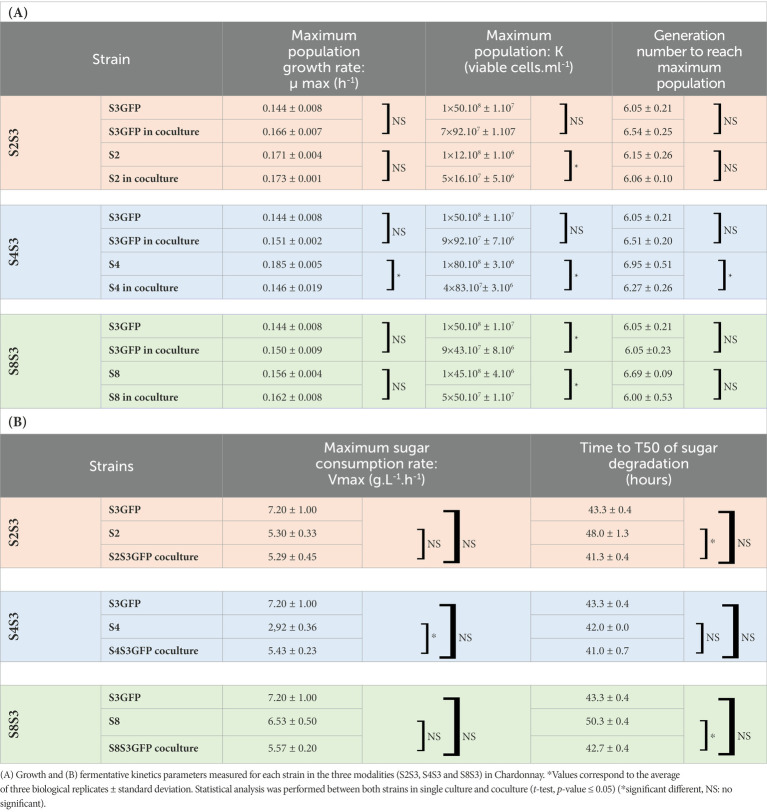

(A) Growth and (B) fermentative kinetics parameters measured for each strain in the three modalities (S2S3, S4S3 and S8S3) in Chardonnay. *Values correspond to the average of three biological replicates ± standard deviation. Statistical analysis was performed between both strains in single culture and coculture (*t*-test, *p*-value ≤ 0.05) (*significant different, NS: no significant).

**Figure 2:**
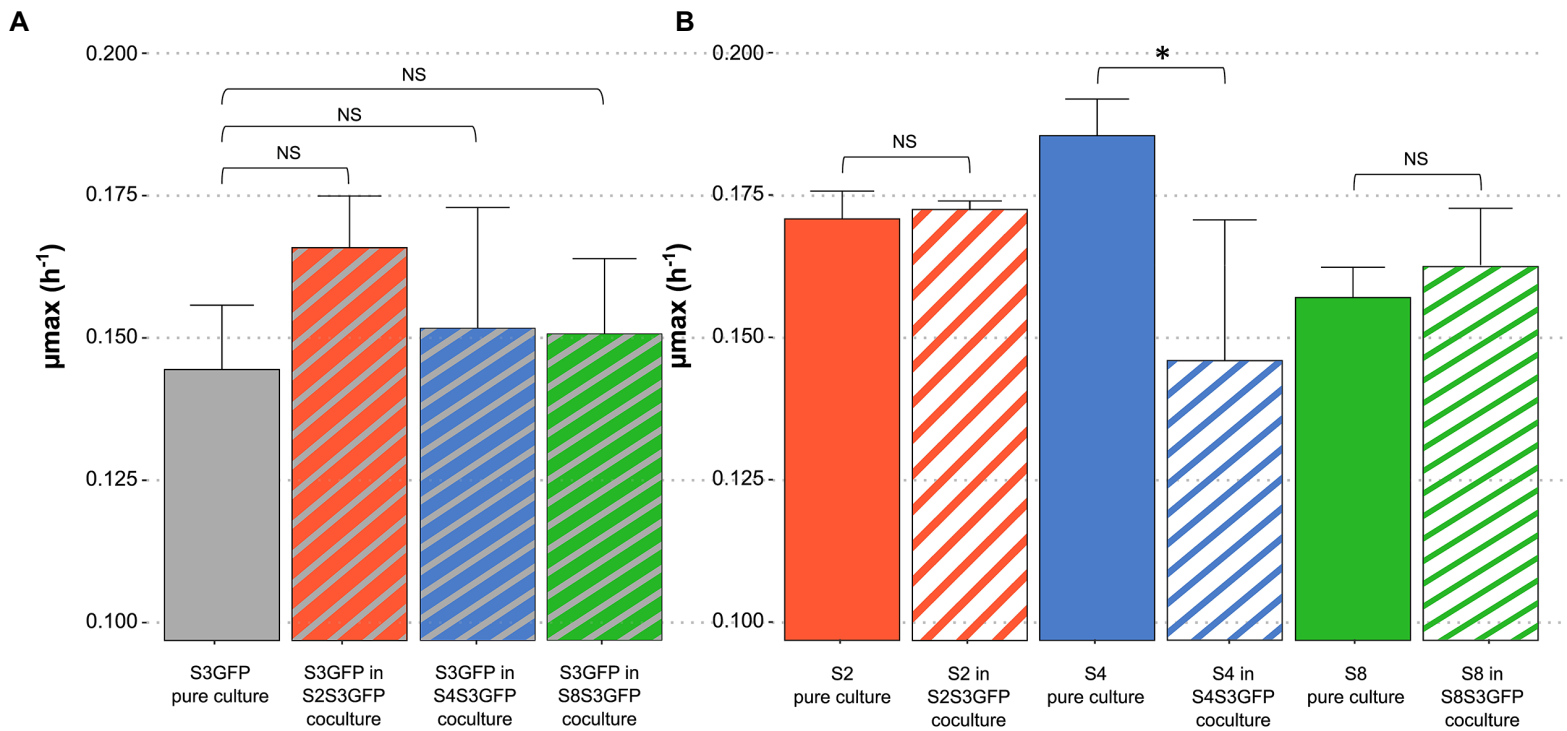
Comparison of maximum growth rate of each strain in pure and coculture. Maximum growth rate of each *S. cerevisiae* strain population (μmax), in Chardonnay, as a function of **(A)** S3GFP in pure culture or in each coculture for each modality: S2S3 (Orange), S4S3 (Blue) and S8S3 (Green) and **(B)** S2, S4 and S8 in pure culture and in each coculture for each modality: S2S3 (Orange), S4S3 (Blue) and S8S3 (Green). μmax was expressed in h^−1^. Values correspond to the average of three biological replicates and error bars represent the standard deviation. Statistical analysis was performed between both pure culture and coculture for each strain involved (*t*-test, value of *p* < 0.05) (NS: non-significant), *significant different.

The maximum growth rate (μ max) was determined for each strain in pure cultures and cocultures for each modality studied (Figure 2). No significant differences were observed when comparing the μmax of S3GFP in pure culture with the μmax of this strain in S4S3GFP coculture. This was also observed for the other two modalities, S2S3 and S8S3. Therefore, the interactions did not impact the μmax of the S3GFP strain. The μmax of pure culture S4 was compared with the μmax of this strain in coculture S4S3GFP and highlighted significant differences. The interactions occurring between the two strains induced a reduction of the μ max of the S4 strain in contrast to modalities S2S3 and S8S3, where this reduction was not observed, with the μmax of S2 and S8 in coculture not differing for each strain in pure culture and thus with no incidence on the μmax.

μmax values were used to calculate the fitness advantage (*m*) of strains in coculture. The m value for the S4S3 modality was close to zero and slightly negative (*m* = −0.04) for S4 vs. S3GFP, indicating similar fitness for both strains in the coculture. This was also noted for the S2S3 and S8S3 modalities, with an *m* of 0.00 and 0.01, respectively.

Considering the maximum population K (Table 2), the addition of the two maximum populations of the two strains S4 and S3GFP in the coculture is the same as each associated pure culture, i.e., 1.50 × 10^8^ viable cells.ml^−1^. However, at the mid-fermentation stage, the population of S4 was half that of S3GFP (Supplementary Figure S3). This modulation of the population could be due to a change in nutrient uptake, particularly of nitrogen. This has been reported in studies that quantified nutrient sources over time and gene expression in pure and cocultures of *S. cerevisiae* and non-*Saccharomyces* (Ruiz et al., 2020). In addition, cell–cell contact could explain a modification of this assimilation (Yashiroda and Yoshida, 2019). Similar growth kinetics were observed for the S2S3 and S8S3 modalities when comparing pure cultures and cocultures. Furthermore, the cocultured strains exhibited equivalent populations during alcoholic fermentation, supporting neutral interactions.

It was also possible to determine the number of generations to reach this maximum population (Table 1). The S4 strain displayed a significantly different number of generations to reach its maximum population of 6.95 and 6.27, when considering the pure culture versus the coculture, respectively. Indeed, the S4 strain was apparently significantly affected in its development within the coculture. Therefore, minor detrimental interactions for S4 could explain this modulation of growth. Contrary to the S2S3 and S8S3 modalities, no difference was observed, indicating that the interactions were neutral.

All the fermentations carried out in Chardonnay must led to a similar ethanol concentration. Other classical oenological parameters such as pH, malic acid, and volatile acidity appeared not impacted by the different modalities of inoculation (pure culture or coculture) (Supplementary Table S2). We also compared the fermentation kinetics of the pure cultures and the cocultures according to two parameters: the maximum rate of sugar degradation and the time to degrade 50% of the initial fermentable sugars. Indeed, it was not possible to dissociate the two strains within the coculture. Thus, the Vmax and T50 of each pure culture were compared to the Vmax and T50 of the associated coculture.

We showed that only the S4S3 coculture showed a significant difference in Vmax compared to the S4 strain in pure culture. For all the other strains, no difference in Vmax was found when considering the pure and cocultures. However, if we examine the data, it appears that the Vmax value (5.43 g.l^−1^.h^−1^) of the coculture was an intermediate of the Vmax of S4 and S3GFP in pure culture and did not differ significantly from the average of the latter (5.06 g.l^−1^.h^−1^) as described previously for the co-inoculation of non*-Saccharomyces* and *S. cerevisiae* (Harlé et al., 2020). Therefore, interactions between two *S. cerevisiae* strains did not affect Vmax. The time to degrade 50% of the initial fermentable sugars (T50) was found to be not significantly different if we consider the pure culture of the S4 strain and the coculture, like for the S8S3 modality. It was therefore observed that the T50 was not negatively impacted by the interactions for these two modalities. On the contrary, for the S2S3 modality, the average of the T50 values of the two strains in culture (45.65 h) was significantly higher than the T50 of the coculture (41.33 h). Thus, combined with the Vmax observations, there was a slowdown in the consumption of sugars during the early stages of fermentation.

The presence of two *S. cerevisiae* strains and the interactions that may occur between them did not confer a fitness advantage to either of the strains present. However, these interactions could induce a modulation of the population, like for the S4S3 modality. Interactions were shown to be strain dependent and confirmed previous observations (Wang et al., 2015). Moreover, it was shown that the fermentation kinetics were not affected by the interactions, in the experimental conditions. However, it is interesting to note that despite interactions that appear neutral at this scale, they can induce a modification of yeast metabolism. Thus, we were interested in assessing the impact of these interactions on the volatile composition of wines.

In a larger study, it was observed that the S3GFP strain exhibited a different metabolism than the wild-type strain although it had similar growth and fermentative kinetics (Bordet et al., 2022). Therefore, for the remainder of the study, the modalities incorporating this S3GFP strain were discarded to exclude the effect of transformation on metabolism at the exometabolome and volatilome scale. In addition, it was desired to get closer to real-world conditions by studying commercialized strains.

### 3.2. Volatilome analysis

The volatilome of wines from pure cultures and cocultures was analyzed. The concentration of 67 volatile compounds determined by GC-FID or GC–MS is reported in Supplementary Table S3.

**Figure 3:**
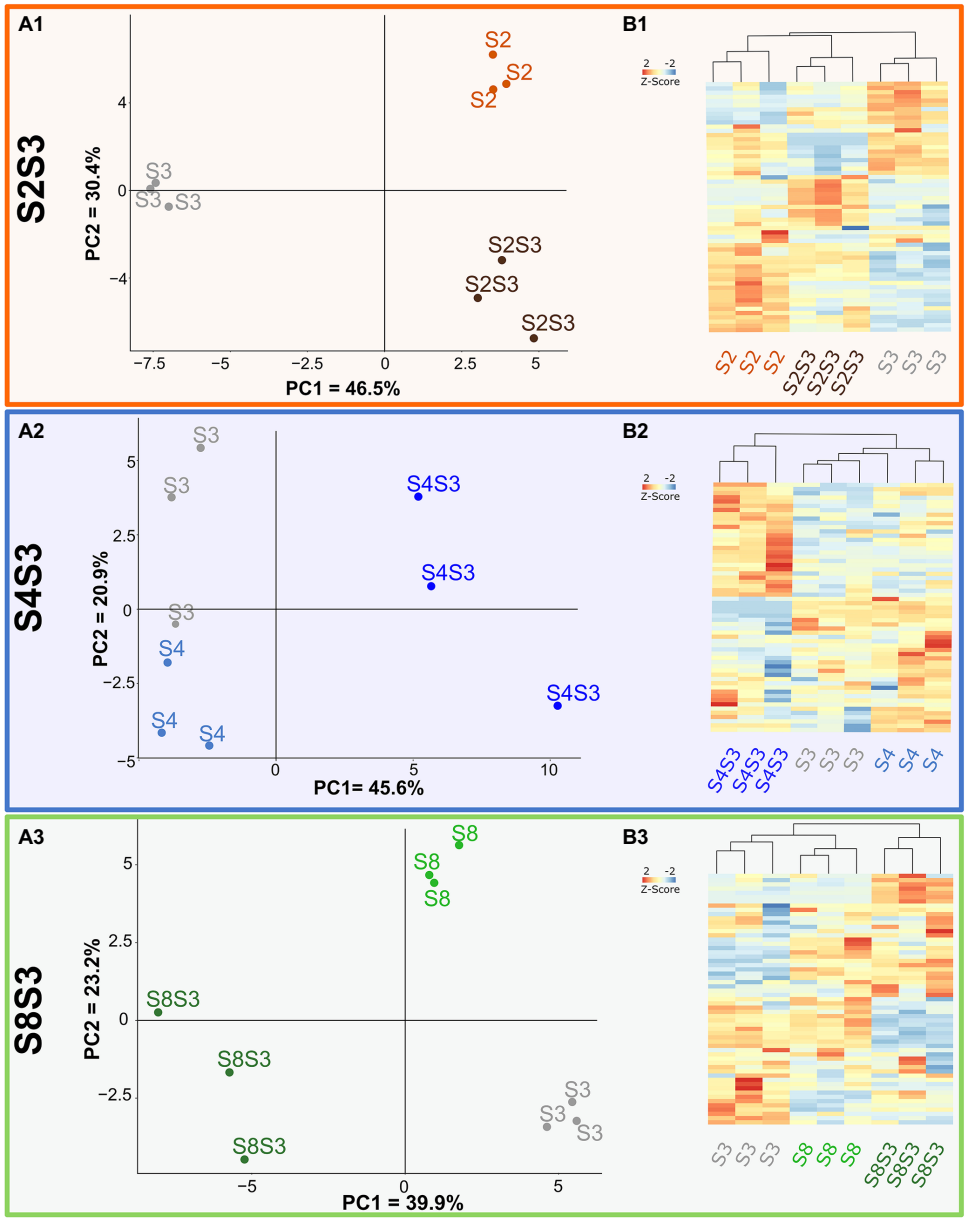
Impact of cocultures on volatilome composition. Volatilome analysis **(A)** PCA of data of Chardonnay wines from pure cultures and coculture for each modality S2S3 (A1), S4S3 (A2), S8S3 (A3). **(B)** Heatmap representing different compounds in pure cultures and cocultures.

Volatile compounds detected in pure and coculture wines for each modality were discriminated using Principal Component Analysis (PCA; Figure 3A). These projections were able to separate the pure culture fermentations from the coculture fermentations for each modality. The strongest effects of the coculture were observed for the S4S3 modalities, shown in Figure 3, A2. As can be seen, the PCA axis 1, explaining 45.6% of the variability data, allowed discrimination of pure cultures from the related cocultures, which demonstrates that the wines produced by the coculture are different to those of the pure cultures. This was also the case for the S8S3 modality. As shown in the A3 plot, the wines made by coculturing were clearly differentiated from those made with pure cultures in the 1^st^ component, which accumulated nearly 40% of the original variance. In the case of the S2S3 modality, however, the A1 plot demonstrates that wines made with S3 in pure culture were the most different. In spite of that, the plot suggests that the volatile composition of the S2S3 coculture is not an intermediate between the two single strains composing it but had a profile closer to S2. These results are a consequence of the fact that S2 strain has a specific volatilome and indicates that the S2 strain contributed significantly to the volatilome of the S2S3 coculture.

One-way ANOVA and then a Tukey HSD test were performed to assess volatile composition differences between the pure culture and co-cutlure within each modality. Compounds that displayed significant differences between pure cultures and cocultures were plotted on HeatMaps (Figure 3B). Modality S4S3 displayed 33 compounds out of 60 detected, showing significant concentration differences between the two pure cultures and the associated coculture. Out of these 33 compounds, 4 belonged to the ester family, 7 were phenols and 6 were part of the fatty acids family. Amino acids and nitrogen compounds are known to be precursors of volatile organic compounds such as acetate esters and therefore involve nitrogen metabolism in this synthesis. Thus, a modulation of the concentration of esters could indicate an impact of interactions in nitrogen metabolism and/or central carbon metabolism. In addition, interestingly, a significant increase of three fatty acids was found in the same wine. Overexpression of these two families of compounds in S4S3 coculture could be related to lipid metabolism, which is also involved in ester synthesis, as described by Saerens et al, (2008). A third family of volatile compounds, i.e., phenols, was impacted during the culture of the two strains. An increase in these compounds was noted for all three modalities, S4S3, S2S3 and S8S3. Thus a variation of the enzymatic activity of decarboxylase (Loscos et al., 2007), leading to the decarboxylation of phenolic acids into vinyl phenols, could be induced by the interactions.

Only the discriminant compounds with an Odour Activity Value reported in Supplementary Table S4 were considered. Of these compounds, hexanoic acid, octanoic acid and acetic acid were detected to have higher concentrations when the two strains, S4 and S3, were brought together. Acetic acid concentration could be modulated by a modification of gene expression. Indeed, Sadoudi et al. (2012) revealed that there was an increase in gene expression in *S. cerevisiae*, encoding enzymes involved in pyruvate dehydrogenase bypass and leading to a modulation of acetic acid concentration in coculture with *M. pulcherrima* (Sadoudi et al., 2012). Octanoic acid was also overproduced by coculture in the S2S3 modality. This saturated acid is known to be involved in fruity notes but mainly as an intermediate of fatty acid ethyl esters. Oxygen deficiency, which can be induced during coculture by competition between yeasts, has been described as leading to the inhibition of fatty acid synthesis combined with an accumulation of medium chain fatty acids (MCFAs) (Dufour et al., 2003). Moreover, these MCFAs were described to be toxic to yeast. Furthermore, modality S4S3 showed only 2 (methionol and nonalactone) and 3 compounds (phenylethanol, vinylphenol and vinylguaiacol) with a similar concentration to strain S4 and S3 in pure culture, respectively, while the other compounds showed significantly different concentrations for the coculture, which confirmed the modification of the volatilome by the interactions within the cocultures initially revealed by the PCA. Thus, the slighlty higher S3 population within the coculture could contribute more to the volatile chemical profile.

β-ionone, norisoprenoid compound and γ-decalactone were found to be significantly less in the S4S3 coculture than for the S2S3 and S8S3 modalities. These aromatic compounds are found as non-volatile, odorless precursors on the grape berry and can be released by enzymatic activity by yeasts like *S. cerevisiae* (Loscos et al., 2010). α-arabinofuranosidase, α-rhamnosidase and β-glucosidase activities were reported for *S. cerevisiae* species according to the precursors present in the must (Sarry and Gunata, 2004; Ugliano et al., 2006). Therefore, co-inoculation could impact enzymatic activity, allowing the release of the compounds. This was shown for the release and modulation mechanisms of thiols (Howell et al., 2006; Anfang et al., 2009; Zott et al., 2011) and could be effective for the enzymes involved in the release of these volatile compounds. In addition, it has been suggested that the content of nitrogenous compounds would also impact norisoprenoïd compound release (Vilanova et al., 2012). Moreover, modality S8S3 exhibited very few volatile compounds, with significantly different concentrations, and above the perception threshold, between the pure and cocultures, suggesting that interactions between S8 and S3 could have no impact on the composition of the odor active volatilome. Finally, for the S2S3 modality most compounds were present at concentrations equivalent to those of the S2 strain in pure culture. This observation is in agreement with the PCA for the S2S3 modality, while the S2 population in the coculture was equivalent to the S3 population. Therefore, considering population dynamics in cocultures, these results indicated that volatile compositions cannot be explained by population dynamics but rather through interactions.

We observed a modulation of the volatile aroma composition under coculture compared to pure cultures, since there was no variation in the population except for the S4S3 modality. This change in the composition of the volatilome was indeed due to interactions. These changes in the volatilome suggest a modification of the aromatic profile of the wines, taking into account the selected expeimental conditions. The sensory profile of end wines is a major issue, but very few studies have been conducted at this scale. Therefore, in this integrative approach, we wanted to evaluate the sensory impact of co-inoculation of two *S. cerevisiae* strains on the selected Chardonnay matrix. In addition, we integrated the mix with a 50/50 ratio (v/v) of post alcoholic fermentation wines of the two pure cultures, equivalent to a blend of wines associated with the coculture for each of the modalities by comparing it with the cocultures, in order to confirm the presence and type of interactions.

### 3.3. Sensory analysis

**Figure 4:**
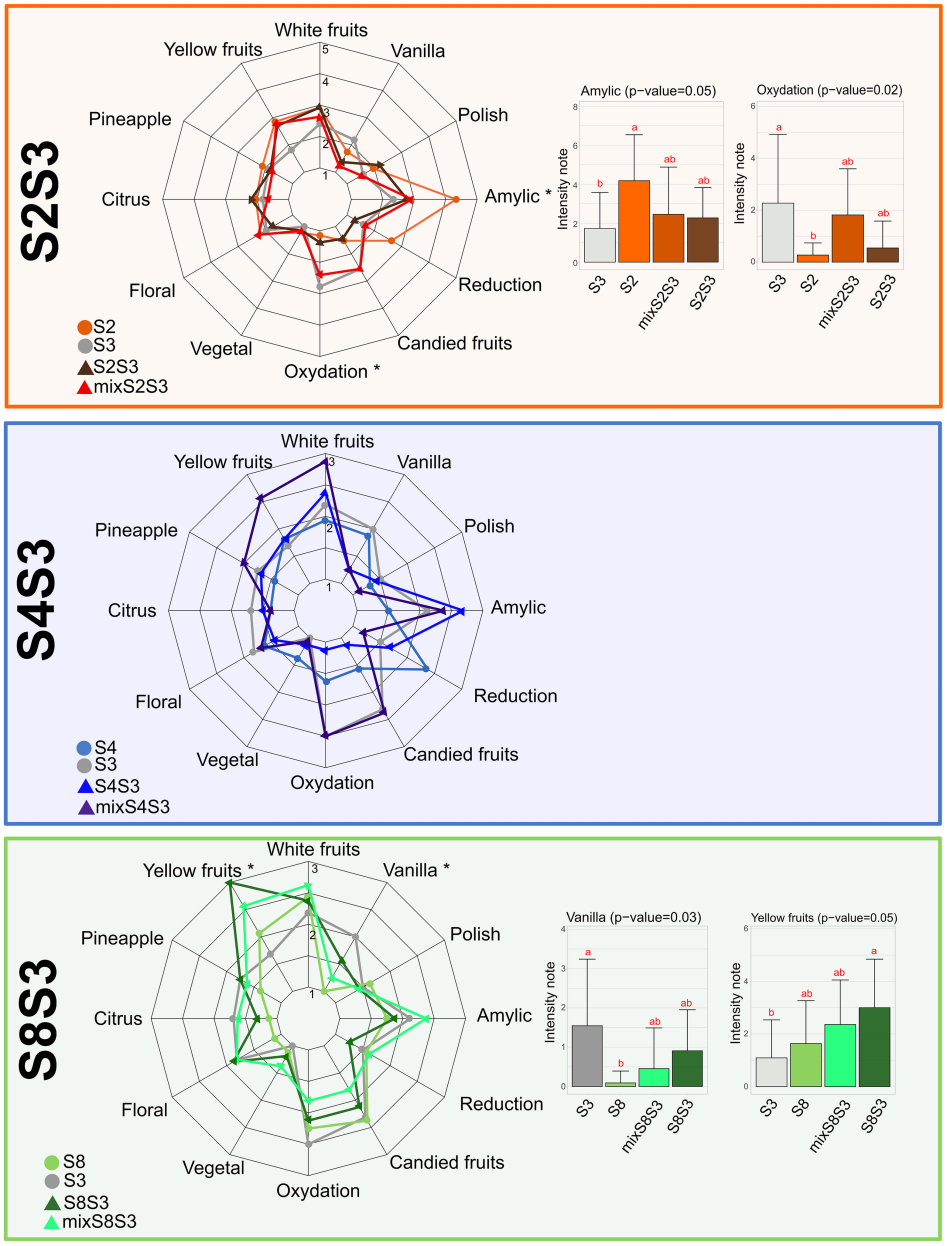
Impact of cocultures on Chardonnay wine sensory profile. Sensory profiles of Chardonnay wines from pure cultures, corresponding cocultures and post-alcoholic fermentation mixes for each modality S2S3 (orange), S4S3 (blue) and S8S3 (green) Histogram of score intensity for attributes showing significant differences (Newman Keuls test, value of *p* ≤ 0.05) between the four wines studied by modality. For modality S4S3 no attribute was significantly different between the different conditions.

At the end of alcoholic fermentation, the wines from the biological replicates of each condition were pooled, sulfited, filtered, bottled, and stored for 6 months. The intensities of each attribute were computed for each sample to determine sensory profiles. Figure 4 shows the average scores for each attribute and each sample for S2S3, S4S3 and S8S3 modalities separately. Regarding the S4S3 modality, it was notable that the S4S3 coculture was distinct from the associated pure cultures. Moreover, the S4S3 mix was found to be very different from the coculture, confirmed by their separation according to axis 1 of the biplot (Supplementary Figure S4). Once again, this confirmed the existence of interactions between the two strains in the co-inoculation process, which in addition modulated the sensory profile of the wines.

The intensity of the oxidative dimension corresponds to oxidation and candied fruit, correlated attributes, and was reduced for the S4S3 modality in the cocultures compared to the pure cultures and the mix, as for S2S3 modality. Thus, in our study, the interaction phenomena would lead to reducing the oxidative dimension of wines. It is interesting to note that the intensity of floral attributes decreased for the coculture, possibly correlating with the decrease of β-ionone synthesis which participates in this characteristic (Loscos et al., 2010). As described previously, isoamyl acetate synthesis participates in bestowing amylic attributes to the S2S3 and S4S3 modalities. On the other hand, methionol concentration in co-inoculating wine participates in reducing this attribute.

For modality S8S3, the coculture appeared to be very close to the mix according to the location on the same side of axis 1 of the PCA biplot, which could confirm the presence of neutral interactions or the lack of interactions. Yellow fruits attribute was the likely result of higher levels of ethyl esters and fatty acids. For the S2S3 modality, significantly different attributes were found between the wines from pure culture fermentations rather than the coculture or the mix. Also, regarding the coculture and the mix, the amylic attribute and oxidation were intermediate in intensity in relation to the pure cultures. The S2 strain in pure culture was also very close to the coculture, which we observed for the other aspects of the study except for polish and amylic. The coculture thus seemed to compensate for the oxidative aspect of the S3 strain. For all the modalities, the mixes did not reflect an intermediate profile between those of the two strains taken separately. This may be explained by interactions between different volatile compounds at the perceptual level.

Thus, we were able to show that the interactions occurring between two *S. cerevisiae* strains led to a modulation of the aromatic profile of wines, in the tested conditions. Indeed, the wines resulting from the coculture appeared different from the mixes and the wines resulting from the pure cultures. Interactions can therefore lead to an interesting modulation of the aromatic profiles of wines. Moreover, the changes observed at the secondary metabolism level, associated with the modification of the volatilome composition, suggest that the primary metabolism would also be affected by the interactions. Therefore, we aimed to use non-targeted metabolomics to report the metabolic changes and describe the types of interactions that took place between two strains of *S. cerevisiae*.

### 3.4. Non volatile metabolome analysis

Chardonnay wine compositions were analyzed by LC-q-TOF-MS. Detected features were extracted from the complete dataset of all the modalities in both positive and negative ionization modes. Of these, the unique masses were excluded for further data processing, resulting in 2054, 2054 and 2,637 features retained for modalities S2S3, S4S3 and S8S3, respectively. Putative annotation was assigned for each extracted feature using an online database (Metlin, KEGG) and the online tool Oligonet. Only features with a putative annotation (annotation Level 3) were retained for subsequent data processing.

**Figure 5:**
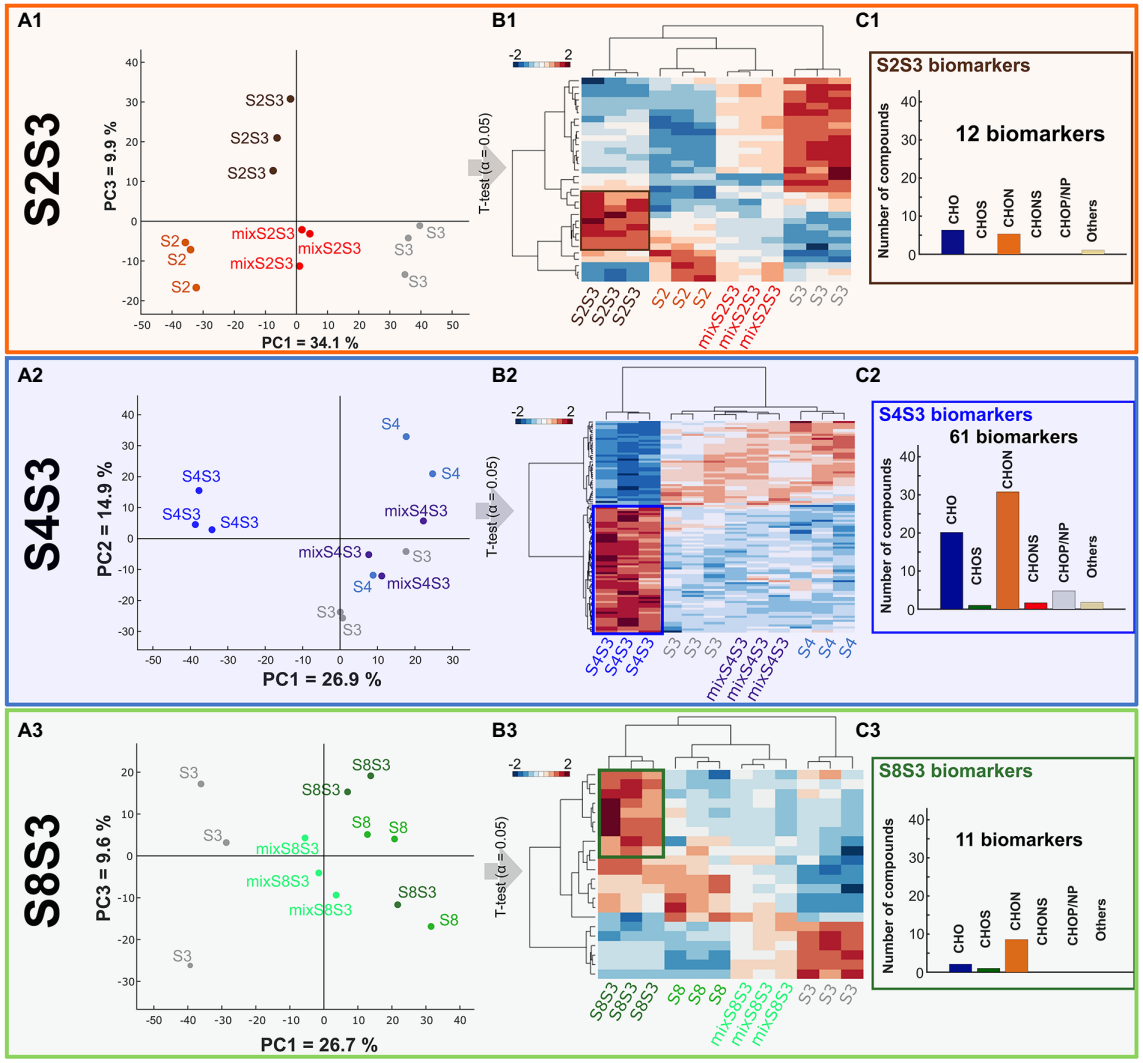
Impact of cocultures on chemical composition. UPLC-q-ToF-MS data **(A)** PCA of data of pure cultures, coculture and post-alcoholic single fermentation Chardonnay wine mixes for each modality S2S3 (orange), S4S3 (blue), S8S3 (green). **(B)** HCA representing extracted significantly different compounds, assessed by *t*-test (value of *p* < 0.05), in pure cultures, cocultures and mixes. **(C)** Histogram proportions of elemental compositions of significantly more intense biomarkers of each coculture.

Principal component analysis (PCA) was performed on LC-q-TOF-MS data for each modality (Figure 5A). All of these representations confirmed the close proximity of biological replicates for a given sample. For each modality, mix samples were localized between the two associated pure cultures, indicating an intermediate composition of pure cultures. Mixes, therefore, corresponded chemically to an equivalent proportion of wines from pure culture yeast fermentations.

Regarding modality S4S3, the coculture was separated from the pure cultures according to PCA Axis 1, explaining 26.9% of the variability data. This important separation supported the previously observed interactions between the two strains S3 and S4 within the coculture. This scheme was also found for the S2S3 modality. Conversely, for modality S8S3 the coculture was not discriminated to the pure culture S8 according to the PCA axis 1. For all the modalities, cocultures were considerably discriminated from the mixes following Axis 1 for modalities S4S3 and S8S3 and following Axis 2 for modality S2S3. Thus, the non-targeted metabolomic study highlighted that these mixes appeared to be different from the cocultures and confirmed interaction mechanisms. Moreover, as observed previously, the impact of the presence of two strains of *S. cerevisiae* varied according to the strain associated with S3.

*T*-tests (α = 0.05) were performed on masses associated with putative annotation (Level 3) for each modality. Putative annotations that displayed significant differences of intensity within each modality were extracted and considered as biomarkers. Of these, 101 were found for modality S4S3, while 32 and 22 were recovered for modalities S2S3 and S8S3, respectively. Thus, there were more differences in the S4S3 modality (between pure and cocultures) than for the other modalities, which is consistent with phenotypic and volatile compound synthesis aspects. A Hierarchical Cluster Analysis (HCA) and a HeatMap of all the biomarkers was established for each modality (Figure 5B). The coculture that was recovered differed from the other conditions, as revealed by the HCA. On the other hand, according to the volatile compound analysis, as shown by the HCA, we noticed that for (S2S3 modality), the S2S3 coculture was closer to the S2 pure culture than the S3 pure culture. By representing the elemental composition of the significantly more intense biomarkers for each coculture of the respective modality (Figure 5C), we observed that S4S3 and S8S3 cocultures were mostly characterized by CHON and CHO compounds, while CHO compounds were more frequent for the S2S3 modality.

LC-q-ToF-MS/MS analysis was performed to confirm the structure of annotated compounds and refine compound identification. Considering the S4S3 modality, among the 101 biomarkers highlighted and associated with putative annotation, 66 were fragmented and 28 annotated according to the Metlin database (Supplementary Table S5) which allows the annotation of metabolites according to their mass and associated fragments from MS/MS analysis (Supplementary Table S6). Of these 28 compounds, 3 were identified using the standard comparison. Leucine was found to be more intense for the S3 pure culture while catechin and quercetin-O-glucoyranoside were significantly increased for the coculture.

Although, only 3 compounds could be identified, fragmentation allowed us to localize biomarkers in the associated metabolic pathways. The annotated biomarkers were grouped into 18 metabolic pathways, classically described in *S. cerevisiae*, for the coculture of the S4S3 modality. We observed that most of the biomarkers were linked to pathways of amino acid metabolism, such as tryptophan and phenylalanine metabolism. Indeed, 8 of these 18 pathways involved in exometabolome changes were associated with this nitrogen metabolism for the S4S3 coculture. This latter metabolism was a major contributor to interaction phenomena. This result confirms a previous study which used a metabolomic approach to reveal the redistribution of fluxes through the central nitrogen metabolism occurring as part of interactions between *Saccharomyces* and non-*Saccharomyces* yeasts (Roullier-Gall et al., 2020). The significance of nitrogen was observed previously using other approaches such as genomics and transcriptomics and reported by Comitini et al. (2021). Moreover, Curiel et al. (2017) reported an increase of expression of genes under control of Nitrogen Catabolism Repression. Genes involved in the utilization of alternative nitrogen sources were also highlighted, during the mixed culture of *T. delbrueckii* and *S. cerevisiae.* On the other hand, an increase in the expression of genes involved in the uptake of nitrogenous compounds from the surrounding environment, for a strain of *S. kudriavzevii* strain was demonstrated to respond to competition by nutrient uptake by *S. cerevisiae,* in case of coculture of *Saccharomyces kudriavzevii* and *S. cerevisiae* (Alonso-del-Real et al., 2019).

In addition, the overexpression of amino acid catabolic intermediates such as phenyl-acetaldoxime was observed for S4S3 coculture, suggesting that as a result of competition for preferential nitrogen compounds, amino acids may be catabolized to substitute them. Moreover, these amino acids were no longer available for the synthesis of volatile compounds such as higher alcohols and associated esters.

Of the S4S3 coculture biomarkers, 30 annotated biomarkers were putatively identified as peptides according to a significant increase in their concentration. During stress situations, including the presence of other microorganisms, significant modifications of protein synthesis are established, inducing an increase in protein production that may be subjected to hydrolysis releasing peptides (Pereira et al., 2021). In addition, the S4 strain has been described as undergoing early autolysis, inducing the release of nitrogenous compounds including peptides (del Barrio-Galán et al., 2016). Also, it is possible that this autolysis is stimulated by the presence of another strain.

High resolution mass spectrometry revealed the modification of the central metabolism related to interactions. It also provided information on the nature of the interactions as non-neutral except for the S8S3 modality. It was observed that the coculture exometabolome is revealed as not being a simple addition of two metabolomes and found as being strain dependent. This was supported by the pattern of the S2S3 modality where the coculture appeared more like the S2 strain in pure culture. The latter would therefore make a significant contribution to the exometabolome of the coculture despite the absence of population variation.

## 4. Conclusion

Most interaction studies have focused on interactions between *Saccharomyces* and non-*Saccharomyces* yeasts according to classical approaches for various applications including modulate aromatic profile of wine. Here we aimed to characterize *S. cerevisiae* interactions and their impact on wine by taking an integrative approach. In the selected experimental conditions, based on growth parameters, the majority of the strains were not affected by the interactions. On the other hand, despite different behaviors at the microbiological point of view, the association of two *S. cerevisiae* strains still induced a modulation of yeast metabolism and of the sensory profile of the wines. Up to thirty volatile compounds were significantly different between the coculture and the associated pure cultures, including 18 relevant wine aroma compounds. Overexpression of linear ethyl esters and corresponding fatty acids was observed. Higher alcohols (including methionol), and their related acetates were affected by coculture. Also, remarkable overexpression of small amounts of volatiles phenols families was noticed. Coculture may make it possible to obtain completely new aromatic expressions attributed to yeast interactions that do not exist in the original pure cultures. The sensory profiles of the cocultures were found to differ from those of the pure cultures regardless of the strain associated with S3. Oxidative dimension was reduced for two cocultures modalities compared to the pure cultures and the mix. Isoamyl acetate synthesis contributes to the high intensity credited to the amylic attributes. Based on the exometabolome, the cocultures were revealed as not being simple additions of two wines represented by mixes, thereby highlighting complex interactions. Thousands of compounds made it possible to pinpoint distinctions within each modality. Cocultures were characterized by significantly more intense CHO and CHON compounds compared to the pure cultures and the associated mix. The latter, involved in central nitrogen metabolism, allowed highlight of metabolic pathways associated with the interactions, including mostly amino acid metabolic pathways. Metabolomics makes it possible to refine the overview of the modifications induced by the, previously observed, interactions at the phenotypic level and from the sensory perspective. Considering the experimental conditions tested, the modulation of the aromatic and chemical profile of wines by interactions between two *S. cerevisiae* strains without altering fermentative properties offers the possibility of using mixed *S. cerevisiae* yeasts to modulate the profile of wines. Indeed, classical approaches such as monitoring of the populations over time are not therefore sufficient to determine and describe the type of interactions that occur. A comprehensive approach by combining different techniques is required to understand them.

## Data availability statement

The original contributions presented in the study are included in the article/Supplementary material, further inquiries can be directed to the corresponding author.

## Author contributions

FBo, RR, HA, CR-G, and AJ-O: conceptualization. FBo, HA, and CR-G: methodology. FBo: validation and writing—original draft. FBo, RR, and FBa: formal analysis. FBo, RR, and CP: investigation. FBo, HA, FBa, RR, RG, AJ-O, VF, CE, CP, JB, and CR-G: writing—review and editing. FBo and RR: visualization. HA, CR-G, and AJ-O: supervision, project administration, and funding acquisition. All authors contributed to the article and approved the submitted version.

## Funding

This work is a part of the METABOLOM (BG0022832.) project supported by the Conseil Régional de Bourgogne-Franche-Comté and the European Union through the PO FEDER-FSE Bourgogne 2014/2020 programs.

## Conflict of interest

The authors declare that the research was conducted in the absence of any commercial or financial relationships that could be construed as a potential conflict of interest.

## Publisher’s note

All claims expressed in this article are solely those of the authors and do not necessarily represent those of their affiliated organizations, or those of the publisher, the editors and the reviewers. Any product that may be evaluated in this article, or claim that may be made by its manufacturer, is not guaranteed or endorsed by the publisher.

## Footnotes

^1^ https://github.com/daniellyz/MXSAlign
