# Supplementary material for: Expanding the diversity of Chardonnay aroma through the metabolic interactions of Saccharomyces cerevisiae cocultures
Source: Front Microbiol. 2023 Feb 9;13:1032842. doi: 10.3389/fmicb.2022.1032842 (PMC9947296; doi:10.3389/fmicb.2022.1032842)
Supplement: Supplementary file 1 [file Data_Sheet_1.docx]

Supplementary Material

**Supplementary Table 1:** Description of each condition with the proportion of each strain. Orange, blue and green colors correspond to S2S3, S4S3 and S8S3 modalities, respectively.

|  | | **Conditions (% of each strain for each condition)** | | | | | | | | | | |
| --- | --- | --- | --- | --- | --- | --- | --- | --- | --- | --- | --- | --- |
|  |  | **S3** | **S3GFP** | **S2** | **S4** | **S8** | **S2 / S3** | **S2 / S3GFP** | **S4 / S3** | **S4 / S3GFP** | **S8 / S3** | **S8 /S3GFP** |
| **Yeast strains** | **S3** | 100 | - | - | - | - | 50 | - | 50 | - | 50 | - |
|  | **S3GFP** | - | 100 | - | - | - | - | 50 | - | 50 | - | 50 |
|  | **S2** | - | - | 100 | - | - | 50 | 50 | - | - | - | - |
|  | **S4** | - | - | - | 100 | - | - | - | 50 | 50 | - | - |
|  | **S8** | - | - | - | - | 100 | - | - | - | - | 50 | 50 |


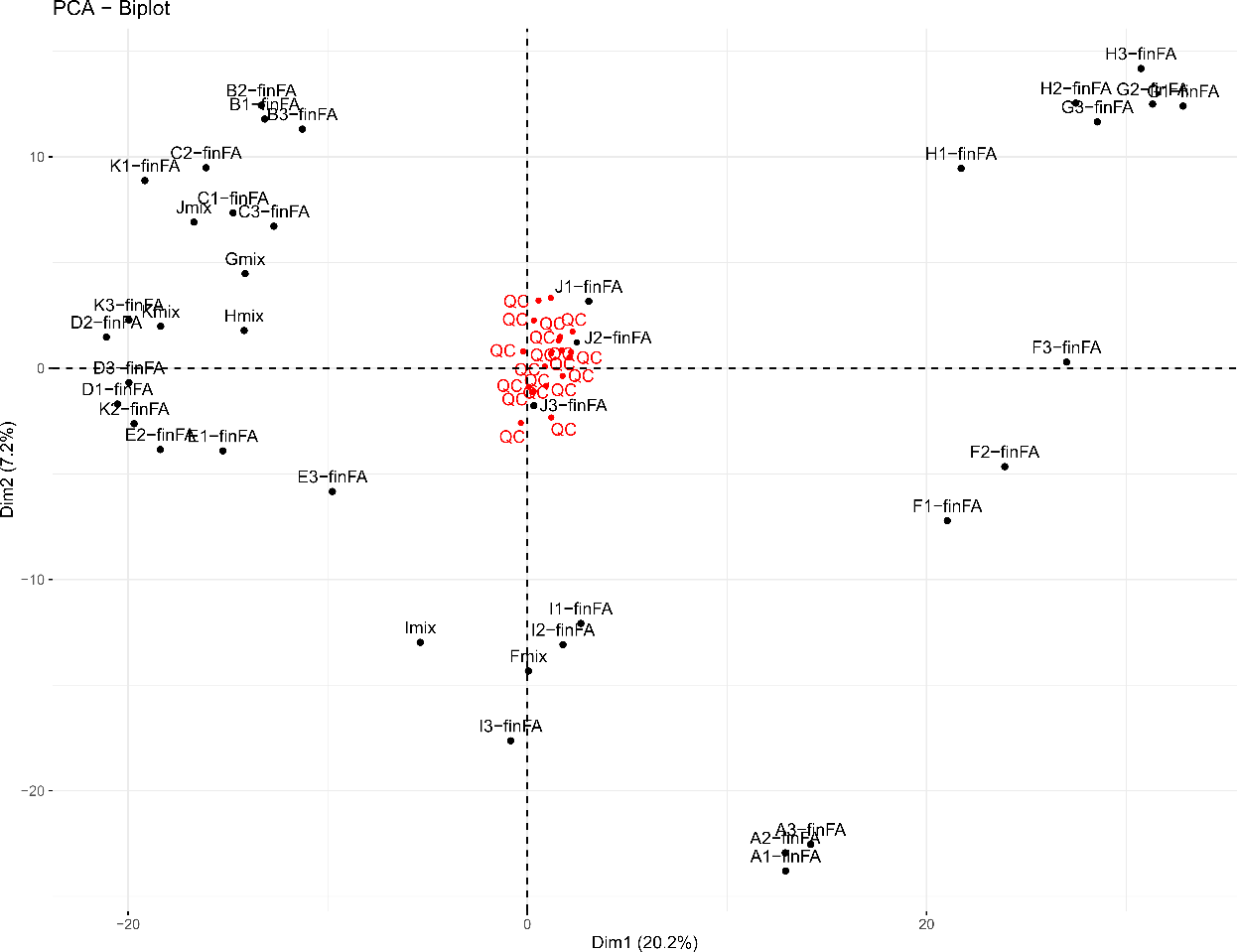


**Supplementary Figure 1 :** Biplot of PCA anlaysis UPLC-QTOF-MS data. QCs are highlighted in red.


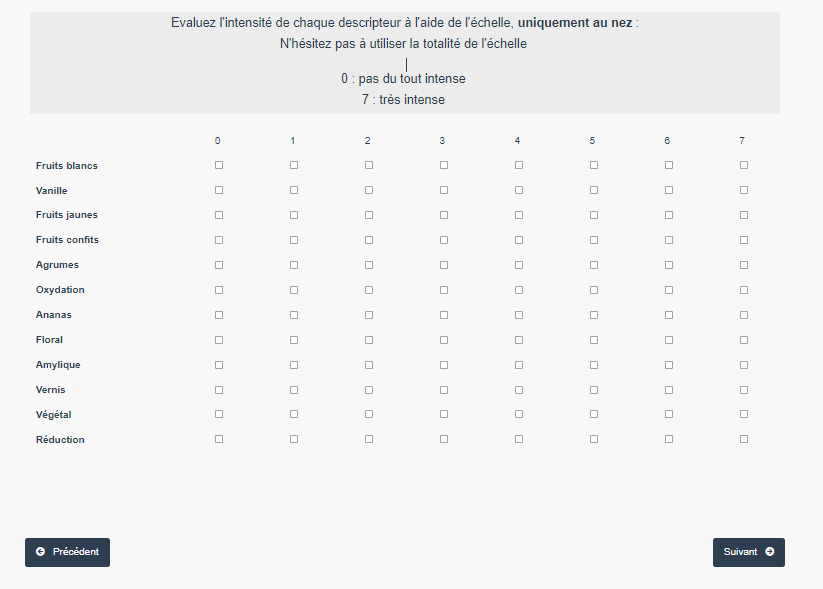


**Supplementary Figure 2 :** Ballot used for the intensity notation on scale (Redjade software)

**Supplementary Table 2:** IRTF physico-chemical characterization of post-fermentation wines Chardonnay. Values correspond to the average of three biological replicates ± standard deviation

|  | **S2** | **S3** | **S4** | **S8** | **S3GFP** | **S2S3** | **S4S3** | **S8S3** | **S2S3GFP** | **S4S3GFP** | **S8S3GFP** |
| --- | --- | --- | --- | --- | --- | --- | --- | --- | --- | --- | --- |
| **Ethanol Vol (%)** | 13,5 ± 0,1 | 14,1 ± 0,17 | 13,5 ± 0,15 | 13,4 ± 0,17 | 13,8 ± 0,15 | 13,77 ± 0,15 | 13,77 ± 0,32 | 13,77 ± 0,15 | 13,8 ± 0,17 | 13,83 ± 0,15 | 13,9 ± 0,2 |
| **pH** | 3,81 ± 0,01 | 3,8 ± 0,02 | 3,85 ± 0,01 | 3,84 ± 0,01 | 3,85 ± 0,01 | 3,86 ± 0,01 | 3,86 ± 0,02 | 3,88 ± 0,01 | 3,82 ± 0,01 | 3,8 ± 0,01 | 3,8 ± 0,02 |
| **AV** | 0,22 ± 0,02 | 0,12 ± 0 | 0,15 ± 0,02 | 0,11 ± 0,04 | 0,08 ± 0,02 | 0,06 ± 0,01 | 0,07 ± 0,02 | 0,06 ± 0,01 | 0,16 ± 0,03 | 0,16 ± 0,02 | 0,13 ± 0,04 |
| **Malic acid (g.L^-1^)** | 3,5 ± 0,1 | 3,4 ± 0 | 3,4 ± 0,06 | 3,57 ± 0,38 | 3,47 ± 0,1 | 3,37 ± 0,15 | 3,4 ± 0,17 | 3,47 ± 0,31 | 3,27 ± 0,12 | 3,23 ± 0,06 | 3,23 ± 0,12 |


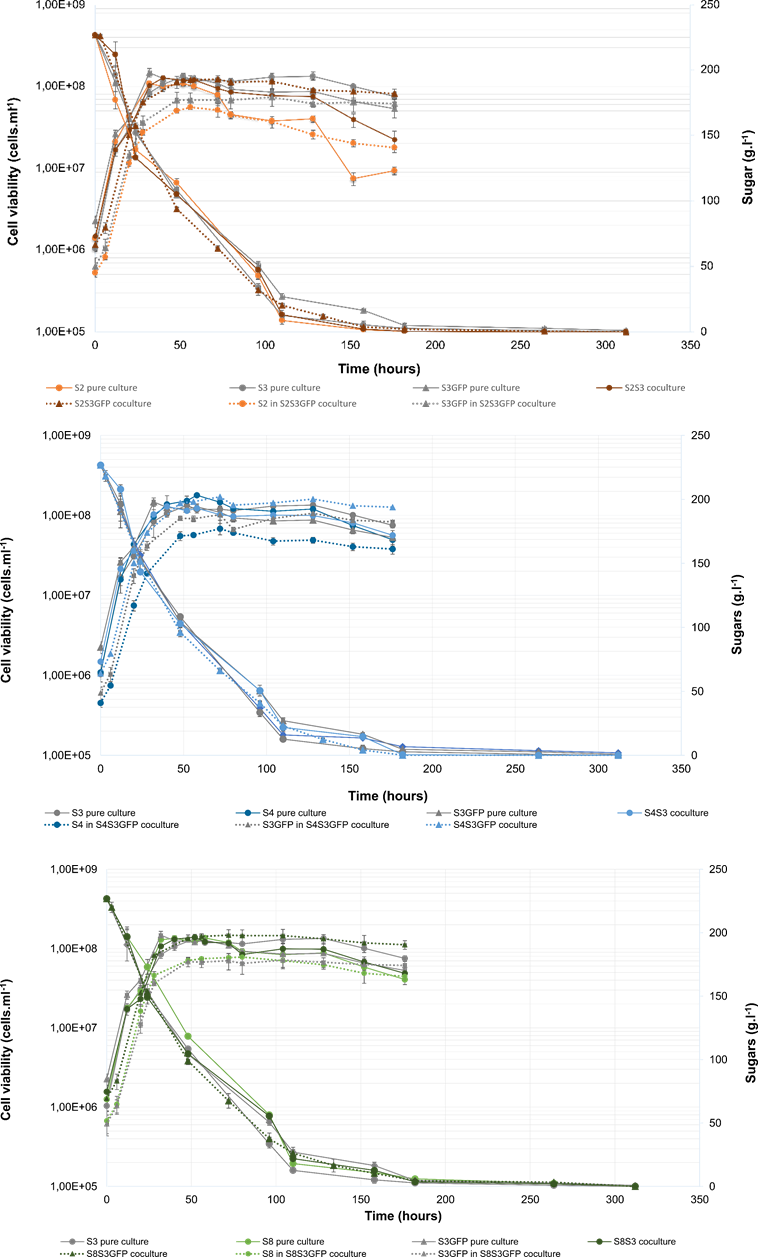
**Supplementary Figure 3:** Fermentation profile comparison in Chardonnay must. Cell viability and sugar consumption were monitored for each condition of *S. cerevisiae* of each modality (S2S3 (orange), S4S3 (blue), S8S3 (green)). Solid curves represent pure culture and dotted curves represent cocultures. For each condition, the experiments were performed in triplicate and the error bars represent the confidence interval.

**Suplemental Table 3:** Table of concentrations of each volatile compound. Values correspond to the average of three biological replicates ± standard deviation (Nd : no detected, <LQ : concentration below the quantification limit), concentrations are expressed in µg.l^-1^.

**Suplemental Table 4:** Table of concentrations of each volatile compound associated to an OAV > 1 with standard deviation and letter (S2S3 (orange), S4S3 (blue), S8S3 (green)), concentrations are expressed in µg.l^-1^

|  | **S3** | **S2** | **S2S3** |
| --- | --- | --- | --- |
| **Ethyl acetate** | 38068.00 ± 2222.00^c^ | 72445.67 ± 1523.11^b^ | 56718.33 ± 1989.11^a^ |
| **Ethyl butyrate** | 111.67 ± 5.56 ^b^ | 116.33 ± 5.78^ab^ | 134.00 ± 7.33^a^ |
| **Isoamyl acetate** | 1672.67 ± 30.22^c^ | 5516.67 ± 630.22^b^ | 3962.00 ± 192.00^a^ |
| **Isoamyl alcohol** | 168006.33 ± 3216.89^b^ | 202958.00 ± 5009.67^a^ | 185724.33 ± 11664.44^ab^ |
| **Methionol** | 1259.33 ± 101.78^b^ | 2618.67 ± 125.78^a^ | 1736.33 ± 243.78^b^ |
| **β-Phenylethanol** | 27324.33 ± 1197.11^b^ | 53859.33 ± 2229.56^a^ | 42970.00 ± 5334.67^a^ |
| **Butyric acid** | 583.00 ± 44.00^b^ | 785.33 ± 30.89^a^ | 705.00 ± 29.33^a^ |
| **Isobutyric acid** | 1438.33 ± 86.89^a^ | 1045.00 ± 62.67^b^ | 1006.67 ± 54.89^b^ |
| **Acetic acid** | 310306.8 ± 15018.8^a^ | 133571.3 ± 20728.5^b^ | 250908.3 ± 60639.4^ab^ |
| **Hexanoic acid** | 3372.67 ± 157.56^b^ | 4513.67 ± 20.44^a^ | 4449.00 ± 95.33^a^ |
| **Octanoic acid** | 4372.00 ± 105.33^b^ | 4624.00 ± 267.33^b^ | 5373.00 ± 189.33^a^ |
| **Phenylethyl acetate** | 266.11 ± 4.38^b^ | 618.83 ± 32.48^a^ | 536.31 ± 52.09^a^ |
| **β-Damascenone** | 2.12 ± 0.02^a^ | 2.04 ± 0.14^ab^ | 1.76 ± 0.04^b^ |
| **β-Ionone** | 0.25 ± 0.01^a^ | 0.24 ± 0.01^a^ | <LQ^b^ |
| **4-Vinylguaiacol** | 503.46 ± 15.04^a^ | 362.16 ± 16.28^b^ | 358.17 ± 13.98^b^ |
| **Ƴ-Decalactone** | 1.33 ± 0.15^a^ | 1.23 ± 0.14^a^ | <LQ^b^ |

|  | **S3** | **S4** | **S4S3** |
| --- | --- | --- | --- |
| **Isoamyl acetate** | 1672.67 ± 30.22^ab^ | 1252.00 ± 115.33^a^ | 2006.67 ± 269.56^b^ |
| **Ethyl butyrate** | 111.67 ± 5.56^ab^ | 90.67 ± 4.44^b^ | 134.33 ± 16.44^a^ |
| **Methionol** | 1259.33 ± 101.78^a^ | 789.00 ± 97.33^b^ | 876.33 ± 48.89^b^ |
| **β-Phenylethanol** | 27324.33 ± 1197.11^a^ | 19343.00 ± 2288.00^b^ | 26647.33 ± 125.78^a^ |
| **Acetic acid** | 310306.67 ± 15018.89^b^ | 288357.33 ± 46135.78^b^ | 429030.67 ± 7431.11^a^ |
| **Isobutyric acid** | 1438.33 ± 86.89^a^ | 1263.33 ± 47.78^ab^ | 1101.67 ± 16.44^b^ |
| **Hexanoic acid** | 3372.67 ± 157.56^b^ | 3209.00 ± 78.00^b^ | 4265.33 ± 214.44^a^ |
| **Octanoic acid** | 4372.00 ± 105.33^b^ | 4339.00 ± 136.00^b^ | 5516.00 ± 341.33^a^ |
| **Phenylethyl acetate** | 266.11 ± 4.38^a^ | 200.47 ± 21.62^b^ | 234.51 ± 6.17^ab^ |
| **β-Ionone** | 0.25 ± 0.01^a^ | 0.23 ± 0.01^a^ | 0.04 ± 0.02^b^ |
| **4-Vinylguaiacol** | 503.46 ± 15.04^a^ | 253.66 ± 7.97^b^ | 479.32 ± 50.82^a^ |
| **4-Vinylphenol** | 743.11 ± 54.5^a^ | 550.87 ± 33.96^b^ | 748.16 ± 49.88^a^ |
| **Ƴ-Decalactone** | 1.33 ± 0,15^a^ | 1.4 ± 0.09^a^ | <LQ^b^ |

|  | **S3** | **S8** | **S8S3** |
| --- | --- | --- | --- |
| **Methionol** | 1259.33 ± 101.78^a^ | 812.00 ± 32.00^b^ | 1232.67 ± 168.44^a^ |
| **Acetic acid** | 310306.67 ± 15018.89^b^ | 412380.00 ± 44452.00^a^ | 329575.00 ± 16870.00^ab^ |
| **Isobutyric acid** | 1438.33 ± 86.89^a^ | 856.67 ± 38.89^c^ | 1122.33 ± 34.22^b^ |
| **Hexanoic acid** | 3372.67 ± 157.56^b^ | 3991.00 ± 107.33^a^ | 4233.33 ± 161.11^a^ |
| **Octanoic acid** | 4372.00 ± 105.33^b^ | 6194.67 ± 217.78^a^ | 5157.67 ± 505.11^ab^ |
| **β-Ionone** | 0.25 ± 0.01^a^ | 0.22 ± 0.02^a^ | 0.05 ± 0.02^b^ |
| **4-Vinylguaiacol** | 503.46 ± 15.04^a^ | 500.59 ± 21.77^a^ | 384.17 ± 19.25^b^ |
| **Ƴ-Decalactone** | 1.33 ± 0.15^a^ | 0.49 ± 0.30^b^ | <LQ ^b^ |


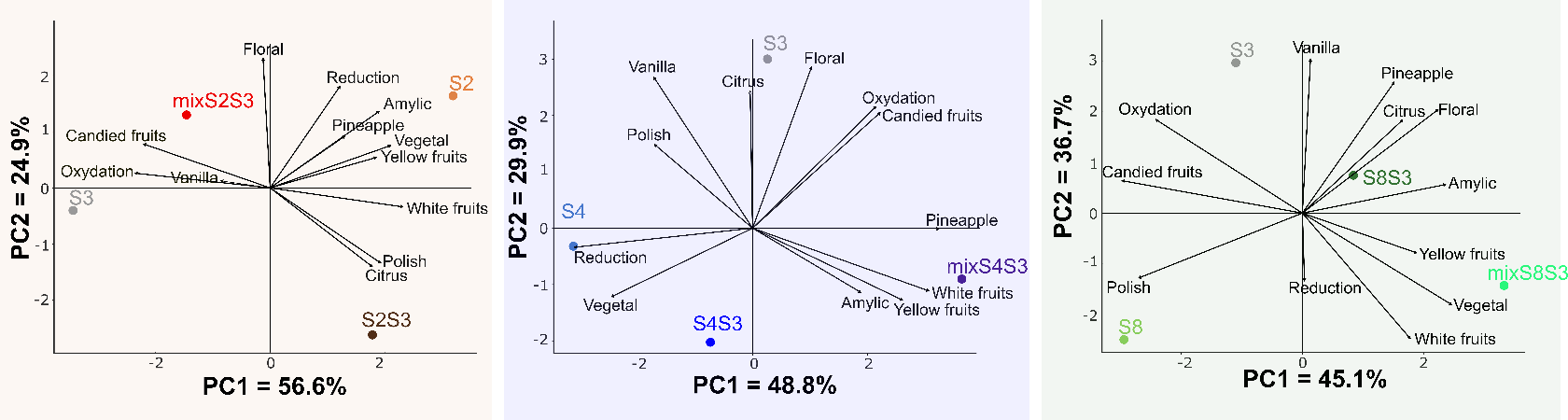


**Suplemental Figure 4 :** Biplot of PCA of Chardonnay wines from pure cultures. corresponding co-cultures and post-alcoholic fermentation mixes for each modality (S2S3 (orange), S4S3 (blue), S8S3 (green))

**Supplementary Table 5:** Data base annoation of fragmented masses for eache condition

| ID | RT(min) | m/ z | MolecularFormula | Database nnotation | Condition |
| --- | --- | --- | --- | --- | --- |
| 497 | 4,64 | 187,13383 | C10H20O3 | 10-Hydroxydecanoic acid; 10-Hydroxydecanoate ; 6-Hydroxy-3,7-dimethyloctanoate | S2S3 |
| 561 | 3,84 | 214,14343 | C11H19NO3 | N-Heptanoylhomoserine lactone | S3 |
| 762 | 5,39 | 215,16491 | C12H24O3 | 12-Hydroxydodecanoic acid; omega-Hydroxydodecanoic acid ; 7-Hydroxydodecanoate | S2S3 |
| 763 | 5,38 | 239,16148 | C12H24O3 | 12-Hydroxydodecanoic acid; omega-Hydroxydodecanoic acid ; 7-Hydroxydodecanoate | S2S3 |
| 764 | 5,65 | 239,16125 | C12H24O3 | 12-Hydroxydodecanoic acid; omega-Hydroxydodecanoic acid ; 7-Hydroxydodecanoate | S2S3 |
| 1065 | 2,29 | 325,0926 | C15H18O8 | 4 -O-beta-D-Glucosyl-cis-p-coumarate ; 4-O-beta-D-Glucosyl-4-hydroxycinnamate ; Bilobalide ; cis-beta-D-Glucosyl-2-hydroxycinnamate; beta-D-Glucosyl-2-coumarinate ; p-Coumaroyl-D-glucose; 1-O-(4-Coumaroyl)-beta-D-glucose; 1-O-(4-Hydroxycinnamoyl)-beta-D-glucose ; trans-beta-D-Glucosyl-2-hydroxycinnamate; beta-D-Glucosyl-2-coumarate | S2S3 |
| 1115 | 4,89 | 221,18931 | C15H24O | (11R)-Dihydroartemisinic aldehyde ; (5R)-Albaflavenol ; (5S)-Albaflavenol ; 10-Hydroxy-alpha-humulene ; 2-trans,6-trans-Farnesal; trans,trans-Farnesal; Farnesal; (2E,6E)-3,7,11-Trimethyl-2,6,10-dodecatrienal; (2E,6E)-Farnesal ; 4-n-Nonylphenol; 4-Nonylphenol; p-Nonylphenol ; Alismol ; Artemisinic alcohol ; Butylhydroxytoluene; BHT; 2,6-Di-t-butyl-4-methylphenol; 2,6-Di-tert-butyl-p-cresol; Butylated hydroxytoluene; 2,6-Bis(1,1-dimethylethyl)-4-methylphenol ; Caryophyllene epoxide; Caryophyllene oxide ; Cyperol ; Germacra-1(10),4,11(13)-trien-12-ol ; Isocyperol ; Nootkatol ; Pentalen-13-ol ; Solavetivol ; [(4S)-4-(5,5-Dimethylcyclohex-1-en-1-yl)-cyclohex-1-en-1-yl]methanol ; alpha-Santalol ; beta-Santalol | S2S3 |
| 1487 | 4,14 | 336,22756 | C18H29N3O3 | Myristyltrimethylaminium bromide; Tetradecyltrimethylammonium bromide; MTAB | S2S3 |
| 138 | 2,45 | 133,08619 | C6H12O3 | (R)-2-Hydroxyisocaproate; D-2-Hydroxyisocaproate; (R)-2-Hydroxy-4-methylpentanoate; D-Leucate ; 6-Hydroxyhexanoic acid; 6-Hydroxyhexanoate ; Ethyl (R)-3-hydroxybutanoate ; Paraldehyde | S2 |
| 249 | 0,71 | 136,07583 | C8H9NO | (E)-Phenylacetaldoxime; (E)-Phenylacetaldehyde oxime ; (Z)-Phenylacetaldehyde oxime; Z-Phenylacetaldoxime ; 2-Phenylacetamide; alpha-Phenylacetamide ; Acetanilide; N-Phenylacetamide; N-Acetylarylamine ; N-Benzylformamide | S3 |
| 1151 | 1,83 | 332,21777 | C15H29N3O5 | LLS ; LTV | S2S3 |
| 1248 | 3,19 | 328,22293 | C16H29N3O4 | LPV | S3 |
| 1506 | 2,5 | 385,24468 | C18H32N4O5 | GLPV ; APVV | S2S3 |
| 1833 | 3,11 | 660,37478 | C28H57N3O12S | DKLVW ; EKVVW ; LLQ ; GATW | S3 |

| 14 | 4,08 | 101,02348 | C4H4O3 | Succinic anhydride; Dihydro-2,5-furandione | S3 |
| --- | --- | --- | --- | --- | --- |
| 30 | 3,74 | 133,06097 | C4H8N2O3 | 3-Ureidopropionate; 3-Ureidopropanoate; beta-Ureidopropionic acid; N-Carbamoyl-beta-alanine ; Asparagine ; D-Asparagine ; Glycylglycine; N-Glycylglycine ; L-Asparagine; 2-Aminosuccinamic acid ; Methylazoxymethanol acetate ; N-Carbamoylsarcosine ; N-Nitroso-N-methylurethane ; N ; GG ; GG | S4S3 |
| 86 | 1,47 | 187,09371 | C5H10N6O2 | Dinitrosopentamethylenetetramine | S4S3 |
| 142 | 0,87 | 132,10232 | C6H13NO2 | (3R)-beta-Leucine; (3R)-3-Amino-4-methylpentanoic acid; (3R)-3-Amino-4-methylvaleric acid ; 6-Aminohexanoate; 6-Aminohexanoic acid; Epsilcapramine; 6-Aminocaproic acid ; D-Isoleucine; (2R,3R)-2-Amino-3-methylvaleric acid ; D-Leucine; D-2-Amino-4-methylvaleric acid ; D-allo-Isoleucine ; Isoleucine ; L-Isoleucine; 2-Amino-3-methylvaleric acid ; L-Leucine; 2-Amino-4-methylvaleric acid; (2S)-alpha-2-Amino-4-methylvaleric acid; (2S)-alpha-Leucine ; L-Norleucine; L-Aminohexanoic acid; L-Aminohexanoate; L-2-Aminohexanoic acid; L-2-Aminohexanoate; (S)-2-Aminohexanoic acid ; L-allo-Isoleucine ; Leucine ; N,N-Diethylglycine ; beta-Alaninebetaine; beta-Alanine betaine; Propiobetaine; Trimethylalanine | S3 |
| 192 | 1,17 | 180,063 | C7H11NO3 | 2-Hydroxyethylclavam; Hydroxyethylclavam ; 3-Methylcrotonylglycine ; Ethadione ; Paramethadione | S4S3 |
| 213 | 2,72 | 191,0846 | C7H14N2O2S | Aldicarb ; Butocarboxim | S4S3 |
| 249 | 0,71 | 136,07583 | C8H9NO | (E)-Phenylacetaldoxime; (E)-Phenylacetaldehyde oxime ; (Z)-Phenylacetaldehyde oxime; Z-Phenylacetaldoxime ; 2-Phenylacetamide; alpha-Phenylacetamide ; Acetanilide; N-Phenylacetamide; N-Acetylarylamine ; N-Benzylformamide | S3 |
| 283 | 4,08 | 197,07796 | C8H14O4 | 2-Propylglutaric acid; 2-PGA ; Dimethoxane ; Dimethyl adipate; Dimethyl hexanedioate ; Suberic acid; Cork acid; 1,8-Octanedioic acid; Octanedioic acid; 1,6-Hexanedicarboxylic acid | S3 |
| 284 | 2,97 | 213,07305 | C8H14O5 | (R)-3-((R)-3-Hydroxybutanoyloxy)butanoate | S4S3 |
| 417 | 1,4 | 230,04237 | C10H9NO4 | 1-Nitro-5,6-dihydroxy-dihydronaphthalene; 1,2-Dihydro-5-nitro-1,2-naphthalenediol ; 2-Formaminobenzoylacetate ; 3-Amino-4,7-dihydroxy-8-methylcoumarin ; 4-(2-Aminophenyl)-2,4-dioxobutanoate | S4S3 |
| 418 | 2,68 | 224,05487 | C10H9NO5 | 3-(1-Carboxyvinyloxy)anthranilate; 2-Amino-3-[(1-carboxyethenyl)oxy]benzoate; 3-Enolpyruvoylanthranilate ; 4-(2-Amino-3-hydroxyphenyl)-2,4-dioxobutanoate ; 4-(2-Amino-5-hydroxyphenyl)-2,4-dioxobutanoate ; 7,8-Dihydro-7,8-dihydroxykynurenate ; N-Malonylanthranilate | S4S3 |
| 425 | 3,22 | 177,05419 | C10H10O4 | 2,4,8-Trihydroxy-1-tetralone ; 5-Hydroxyconiferaldehyde ; 6-Hydroxymellein ; Dimethyl phthalate ; Ferulate; Ferulic acid; 4-Hydroxy-3-methoxycinnamic acid; 3-Methoxy-4-hydroxy-trans-cinnamate; 4-Hydroxy-3-methoxycinnamate ; Isoferulic acid ; Kakuol; Kakoul ; Methyl caffeate ; Scytalone; 3,4-Dihydro-3,6,8-trihydoroxy-1(2H)-napthalenone | S4S3 |
| 478 | 4,45 | 155,14273 | C10H18O | (+)-Borneol; d-Borneol; Borneocamphor; endo-2-Bornanol; Sumatra camphor; (1R,2S,4R)-(+)-Borneol ; (+)-Isomenthone ; (+)-Linalool; (S)-(+)-Linalool; (3S)-Linalool ; (+)-Menthone ; (+)-Thujan-3-ol ; (+)-trans-Piperitenol ; (+)-trans-Pulegol ; (-)-Borneol; l-Borneol; (1S,2R,4S)-(-)-Borneol; Linderol ; (-)-Isomenthone; l-Isomenthone ; (-)-Linalool; (R)-(-)-Linalool; (3R)-Linalool ; (-)-Menthone; l-Menthone; p-Menthan-3-one ; (-)-alpha-Terpineol; (L)-alpha-Terpineol ; (-)-endo-Fenchol; (-)-alpha-Fenchyl alcohol ; (1R,2R,4R)-Dihydrocarveol; (-)-Dihydrocarveol ; (1R,2R,4S)-Iso-dihydrocarveol ; (1R,2S,4R)-Neo-dihydrocarveol ; (1R,2S,4S)-Neoiso-dihydrocarveol ; (1S,2R,4R)-Neoiso-dihydrocarveol ; (1S,2R,4S)-Neo-dihydrocarveol ; (1S,2S,4R)-Iso-dihydrocarveol ; (1S,2S,4S)-Dihydrocarveol; (+)-Dihydrocarveol ; (R)-(+)-Citronellal ; (R)-(+)-alpha-Terpineol; (+)-alpha-Terpineol ; (S)-(-)-Citronellal; (S)-3,7-Dimethyloct-6-enal ; 1,4-Cineole; 1,4-Epoxy-p-menthane ; 1,8-Cineole; 1,8-Cineol; Eucalyptol; 1,3,3-Trimethyl-2-oxabicyclo[2.2.2]octane ; 8-p-Menthen-2-ol; 1,6-Dihydrocarveol; 6-Methyl-3-isopropenylcyclohexanol; Menth-8-en-2-ol ; Borneol ; Chrysanthemol ; Citronellal ; Geraniol ; Linalool; 3,7-Dimethylocta-1,6-dien-3-ol; (RS)-Linalool ; Nerol; (Z)-3,7-Dimethylocta-2,6-dien-1-ol ; Sabinene hydrate ; Terpineol-4; Terpinen-4-ol ; alpha-Terpineol; dl-alpha-Terpineol ; beta-Terpineol ; gamma-Terpineol | S4S3 |
| 496 | 5,67 | 173,15307 | C10H20O2 | Decanoic acid; Decanoate; Decylic acid; n-Capric acid ; Ethyl octanoate; Ethyl caprylate ; Isoamyl isovalerate ; p-Menthane-3,8-diol; (-)-3,8-p-Menthanediol | S4S3 |
| 568 | 2,81 | 213,15945 | C11H20N2O2 | 1,4 -Bipiperidine-1 -carboxylic acid; [1,4 -Bipiperidine]-1 -carboxylic acid | S3 |
| 586 | 2,54 | 249,12594 | C11H21O4P | MV | S4 |
| 594 | 2,78 | 231,17013 | C11H22N2O3 | LV | S4 |
| 595 | 3,07 | 231,17007 | C11H22N2O3 | LV | S3 |
| 711 | 3,17 | 227,1751 | C12H22N2O2 | 1,8-Diazacyclotetradecane-2,9-dione; Cyclo(L-leucyl-L-leucyl); (3S,6S)-3,6-Diisobutylpiperazine-2,5-dione | S4S3 |
| 759 | 2,59 | 261,18079 | C12H24N2O4 | Carisoprodol | S3 |
| 760 | 3,07 | 261,18076 | C12H24N2O4 | Carisoprodol | S4 |
| 764 | 5,65 | 239,16125 | C12H24O3 | 12-Hydroxydodecanoic acid; omega-Hydroxydodecanoic acid ; 7-Hydroxydodecanoate | S4S3 |
| 794 | 2,71 | 214,08587 | C13H11NO2 | Benzyl nicotinate; Nicotinic acid benzyl ester ; Fenamic acid; Diphenylamine-2-carboxylic acid; DPC ; Salicylanilide | S4S3 |
| 846 | 2,68 | 207,13766 | C13H20O3 | (+)-7-Isomethyljasmonate ; (+; -)-6-Hydroxy-3-oxo-alpha-ionol; Vomifoliol ; (6S,9R)-Vomifoliol; (6S,9R)-6-Hydroxy-3-oxo-alpha-ionol ; Methyl jasmonate; (-)-Methyl jasmonate | S4S3 |
| 905 | 3,3 | 229,08562 | C14H12O3 | 2-Hydroxy-4-methoxybenzophenone; Oxybenzone ; 3,4-Dihydro-8,9-dihydroxy-1(2H)-anthracenone ; 4,4 ,6-Trimethylangelicin ; 4-Isonicotinoylnicotinamide; 4-INN ; 5,6-Dehydrokawain ; Benzylparaben; Benzyl parahydroxybenzoate; Benzyl 4-hydroxybenzoate ; Resveratrol; 3,4 ,5-Trihydroxystilbene; trans-Resveratrol ; Seselin ; Trioxsalen; Trioxysalen; 4,8,5 -Trimethylpsoralen ; Xanthyletin | S4S3 |
| 919 | 2,06 | 263,13964 | C14H18N2O3 | Methohexital ; Physovenine | S4 |
| 928 | 2,13 | 265,15453 | C14H20N2O3 | Feruloylputrescine; Subaphyllin ; PF | S4 |
| 970 | 1,54 | 336,15838 | C14H26NO6P | MSV ; CLT | S3 |
| 971 | 4,45 | 287,19636 | C14H26N2O4 | N-Acetyl-leucyl-leucine ; FV | S4S3 |
| 1046 | 1,91 | 291,08645 | C15H14O6 | (+)-Catechin; D-Catechin; Cyanidanol; (2R-trans)-2-(3,4-Dihydroxyphenyl)-3,4-dihydro-2H-1-benzopyran-3,5,7-triol; (2R,3S)-Catechin; (2R,3S)-(+)-Catechin; Cianidanol ; (+)-Epicatechin; ent-Epicatechin ; (-)-Catechin ; (-)-Epicatechin ; 2,8-Dihydroxy-3,4,7-trimethoxydibenzofuran ; Catechin; (+; -)-Catechin ; Fisetinidol-4beta-ol; (-)-Mollisacacidin; Fisetinin-3,4-diol; 5-Deoxyleucocyanidin ; Isoplumericin ; Luteoforol; 3-Deoxyleucocyanidin ; Mikanolide ; Plumericin ; Ptaeroglycol ; alpha-Cotonefuran ; cis-3,4-Leucopelargonidin | S4S3 |
| 1095 | 2,39 | 295,16515 | C15H22N2O4 | LY | S3 |
| 1151 | 1,83 | 332,21777 | C15H29N3O5 | LLS ; LTV | S3 |
| 1349 | 1,79 | 474,25524 | C17H27N15O2 | EKPT ; DLLN ; GG ; DLQ ; GAV ; ELN ; GGV ; EQ ; GAVV ; DGGLL ; ADGLV ; AADVV ; EGGLV ; AEGVV ; AAAEL ; ALPSS ; GLPST ; APSTV ; GPTTV | S3 |
| 1365 | 1,1 | 371,22892 | C17H30N4O5 | GPVV ; AALP | S4 |
| 1366 | 1,62 | 371,22859 | C17H30N4O5 | GPVV ; AALP | S4S3 |
| 1528 | 3,67 | 358,26956 | C18H35N3O4 | Leucyl-leucyl-norleucine LLL | S4 |
| 1548 | 5,72 | 300,28903 | C18H37NO2 | 3-Dehydrosphinganine; 3-Dehydro-D-sphinganine ; Palmitoylethanolamide; Palmidrol ; Sphingosine; Sphingenine; Sphingoid; Sphing-4-enine | S4S3 |
| 1558 | 5,32 | 318,29953 | C18H39NO3 | Phytosphingosine; 4-D-Hydroxysphinganine | S4S3 |
| 1616 | 3,66 | 388,24343 | C19H37N3OS2 | GRR | S4 |
| 1690 | 2,99 | 465,10239 | C21H20O12 | 6-Hydroxyluteolin 7-glucoside ; Bracteatin 6-O-glucoside; Bracteatin 6-O-beta-D-glucoside ; Gossypetin 8-rhamnoside ; Hyperin; Quercetin 3-galactoside ; Myricitrin; Myricetin 3-O-rhamnoside ; Quercetin 3-O-glucoside; Isoquercitrin; 2-(3,4-Dihidroxyphenyl)-3-(beta-D-glucofuranosyloxy)-5,7-dihydroxy-4H-1-benzopyran-4-one; Isotrifoliin ; Quercimeritrin; Quercetin 7-O-beta-D-glucoside ; CCCH | S4S3 |
| 1746 | 3,14 | 472,25499 | C23H37NO9 | GLPW ; APVW | S4S3 |
| 1804 | 1,49 | 615,26201 | C26H49O10P3 | CFKMS ; CCKVY ; ACKMY ; FFGMN ; GG ; ACFFQ ; GA | S4S3 |
| 1833 | 3,11 | 660,37478 | C28H57N3O12S | DKLVW ; EKVVW ; LLQ ; GATW | S3 |

| 14 | 4,08 | 101,02348 | C4H4O3 | Succinic anhydride; Dihydro-2,5-furandione | S3 |
| --- | --- | --- | --- | --- | --- |
| 213 | 2,72 | 191,0846 | C7H14N2O2S | Aldicarb ; Butocarboxim | S8 |
| 256 | 3,43 | 122,09692 | C8H11N | 1-Phenylethylamine; alpha-Phenylethylamine; alpha-Methylbenzylamine ; 2,4-Xylidine; 2,4-Dimethylaniline; 2,4-DMA ; 2,5-Xylidine; 2,5-Dimethyl-benzenamine ; 2,6-Dimethylaniline; 2,6-DMA; 2,6-Xylidine ; N,N-Dimethylaniline; Dimethylaminobenzene; N,N-Dimethylbenzenamine ; N-Ethylaniline; N-Ethylbenzenamine ; Phenethylamine; 2-Phenylethylamine; beta-Phenylethylamine; Phenylethylamine | S3 |
| 283 | 4,08 | 197,07796 | C8H14O4 | 2-Propylglutaric acid; 2-PGA ; Dimethoxane ; Dimethyl adipate; Dimethyl hexanedioate ; Suberic acid; Cork acid; 1,8-Octanedioic acid; Octanedioic acid; 1,6-Hexanedicarboxylic acid | S3 |
| 321 | 2,43 | 163,03871 | C9H6O3 | 4-Hydroxycoumarin ; Umbelliferone; 7-Hydroxycoumarin | S8S3 |
| 349 | 4,4 | 182,08059 | C9H11NO3 | (S)-beta-Tyrosine ; 3-Amino-3-(4-hydroxyphenyl)propanoate; beta-Tyrosine ; 3-Hydroxy-L-phenylalanine; 3-Tyrosine ; D-Tyrosine; (R)-3-(p-Hydroxyphenyl)alanine; (R)-2-Amino-3-(p-hydroxyphenyl)propionic acid ; L-Tyrosine; (S)-3-(p-Hydroxyphenyl)alanine; (S)-2-Amino-3-(p-hydroxyphenyl)propionic acid; Tyrosine ; L-threo-3-Phenylserine ; N-Hydroxy-L-phenylalanine; 2-(Hydroxyamino)-3-phenylpropanoate ; Tyrosine; 3-(p-Hydroxyphenyl)alanine; 2-Amino-3-(p-hydroxyphenyl)propionic acid ; gamma-Hydroxy-3-pyridinebutanoate | S8S3 |
| 390 | 1,29 | 203,13883 | C9H18N2O3 | AL | S8S3 |
| 545 | 5,1 | 210,11171 | C11H15NO3 | Anhalamine; N-Demethylanhalidine ; Propoxur; Aprocarb; 2-Isopropoxyphenyl N-methylcarbamate ; Tyr-OEt ; p-Lactophenetide; 4 -Ethoxylactanilide; Lactylphenetidin | S8S3 |
| 762 | 5,39 | 215,16491 | C12H24O3 | 12-Hydroxydodecanoic acid; omega-Hydroxydodecanoic acid ; 7-Hydroxydodecanoate | S8 |
| 1506 | 2,5 | 385,24468 | C18H32N4O5 | GLPV ; APVV | S8 |
| 1587 | 5,1 | 336,21628 | C19H29NO4 | Ankorine | S8S3 |
| 1651 | 2,36 | 424,2074 | C20H29N3O7 | Amicoumacin A ; ELY | S8 |
| 1679 | 2,12 | 457,26483 | C20H40O11 | DLLP ; ELPV | S8S3 |
| 2021 | 2,52 | 298,52004 | C49H80O12S | Chlorophyll a | S8S3 |

**Supplementary Table 6:** Table of extracted, annotated and identified metabolites of Chardonnay wine fermented by each single strain, coculture or strains mixed for each modality S2S3 (orange), S4S3 (blue), S8S3 (green).

| **Modalities** | **Number of biomarkers with tentative structure (Level 3)** | **Fragmented biomarkers** | **Fragmented biomarkers (putative identification, Level 2)** | **Validated Identification (Level 1)** |
| --- | --- | --- | --- | --- |
| **S2** | 3 | 2 | 1 | - |
| **S3** | 17 | 13 | 2 | - |
| **mixS2S3** | - | - | - | - |
| **S2S3** | 12 | 11 | 7 | - |
|  |  |  |  |  |
| **S4** | 19 | 13 | 4 | - |
| **S3** | 21 | 15 | 6 | 1 |
| **mixS4S3** | - | - | - | - |
| **S4S3** | 61 | 38 | 18 | 2 |
|  |  |  |  |  |
| **S8** | 5 | 5 | 3 | - |
| **S3** | 6 | 5 | 3 | - |
| **mixS8S3** | - | - | - | - |
| **S8S3** | 11 | 10 | 5 | - |
